# Combination therapy with nisin, urolithin B, and vincristine exhibits synergistic antiproliferative and pro-apoptotic effects against human lymphoma cells: evidence from proteomics

**DOI:** 10.3389/fimmu.2025.1664468

**Published:** 2025-09-23

**Authors:** Ahmad K. Al-Khazaleh, Muhammad A. Alsherbiny, Dennis Chang, Gerald Münch, Deep Jyoti Bhuyan

**Affiliations:** ^1^ NICM Health Research Institute, Western Sydney University, Penrith, NSW, Australia; ^2^ Pharmacognosy Department, Faculty of Pharmacy, Cairo University, Cairo, Egypt; ^3^ Freedman Foundation Metabolomics Facility, Innovation Centre, Victor Chang Cardiac Research Institute, Sydney, NSW, Australia; ^4^ Pharmacology Unit, School of Medicine, Western Sydney University, Campbelltown, NSW, Australia; ^5^ School of Science, Western Sydney University, Penrith, NSW, Australia

**Keywords:** lymphoma, vincristine, nisin, urolithin B, synergy, proteomics, apoptosis

## Abstract

Lymphoma remains a significant global health burden, necessitating innovative, targeted therapeutic strategies. Nisin (N), a bacteriocin produced by *Lactococcus lactis*, has demonstrated antimicrobial and anticancer properties through membrane disruption and apoptotic induction. Urolithin B (UB), a gut microbiota-derived metabolite of ellagitannins, has shown anti-inflammatory and antiproliferative activities in various cancer models. Vincristine (Vinc), a common anti-lymphoma drug, disrupts microtubule formation, leading to cell cycle arrest and apoptosis in cancer cells. This study explored the antiproliferative and pro-apoptotic effects of a triple combination therapy comprising N, UB, and Vinc against human lymphoma cell lines (HKB-11 and Hs 313.T). This study systematically evaluated the synergistic efficacy of both monotherapy and dual and triple combinations and molecular mechanisms using Alamar Blue viability assays, combination index (CI) modelling, reactive oxygen species (ROS) quantification, annexin V/7-AAD flow cytometry, and bottom-up label-free proteomics. The potential cytotoxicity of the combination on normal stromal HS-5 cells was also assessed using the Alamar Blue assay. The N: UB: Vinc combination at 2240: 210: 0.94 µM demonstrated potent synergy (CI values 0.31–0.50 at IC_90_ - IC_95_) and induced near-complete growth inhibition (> 99%) in both lymphoma cell lines with reduced toxicity (42.09 ± 1.21% viability) toward normal stromal HS-5 cells. ROS analysis revealed significant oxidative stress, while flow cytometry confirmed enhanced apoptosis (p < 0.0001) in the combination groups. Proteomic profiling of the combination N: UB: Vinc at 2450.94 µM uncovered distinct molecular responses, including upregulation of MAP1LC3B2 (Log_2_FC = 1.4), GMNN (Log_2_FC = 1.3), and SLC38A2 (Log_2_FC = 1.5), promoting apoptosis, cell cycle regulation, and mTOR signaling inhibition. Concurrently, key oncogenic and metabolic proteins were downregulated, including NNMT (Log_2_FC = –2.9), PLTP (Log_2_FC = –2.5), and CYP4X1 (Log_2_FC = –2.0), which implicated the suppression of MAPK-Akt signaling, ferroptosis activation, and lipid metabolism disruption. These results established a mechanistic rationale for combining postbiotics such as N and UB with standard chemotherapy, highlighting a promising avenue for safer and more effective lymphoma management in the future.

## 1 Introduction

Lymphoma encompasses a diverse group of hematological malignancies arising primarily from lymphocytes, presenting considerable heterogeneity in clinical behavior and therapeutic responses. It is broadly categorized into Hodgkin lymphoma (HL) and non-Hodgkin lymphoma (NHL), with NHL accounting for approximately 90% of all lymphoma cases (1, 2). Despite therapeutic advances and improvements in early diagnosis, lymphoma remains a significant contributor to cancer-related morbidity and mortality worldwide. Standard treatment options include chemotherapy, immunotherapy, targeted therapy, and radiation. However, chemotherapy remains the cornerstone of management, particularly Vinc-containing regimens such as CHOP (cyclophosphamide, doxorubicin, Vincristine (Vinc), prednisone) for NHL. Unfortunately, these treatments are often accompanied by severe adverse effects, notably peripheral neuropathy, cardiotoxicity, and myelosuppression, which negatively affect patient outcomes and quality of life (1–3).

Vinc, a vinca alkaloid derived from *Catharanthus roseus*, exerts its antineoplastic effects primarily by disrupting microtubule polymerization, thereby inducing mitotic arrest and apoptosis in rapidly dividing cancer cells (4). Despite its efficacy, Vinc use is significantly limited by dose-dependent peripheral neuropathy, characterized by pain, numbness, and functional impairments that can persist long after treatment cessation (3, 5). Such neurotoxicity underscores the necessity for innovative strategies capable of enhancing therapeutic efficacy while reducing chemotherapy-associated toxicities.

An emerging strategy to treat cancer involves combining conventional chemotherapeutics with natural bioactive agents and gut microbial metabolites (postbiotics). This integrative approach aims to exploit additive or synergistic anticancer mechanisms, potentially allowing for dose reductions and subsequently diminishing treatment-associated adverse effects (6, 7). Among natural compounds, Nisin (N), an antimicrobial peptide produced by *Lactococcus lactis*, has garnered significant interest due to its potential antiproliferative properties through mechanisms such as induction of apoptosis via pore formation in cellular membranes, oxidative stress induction, and immune modulation (8, 9). Likewise, Urolithin B (UB), a gut microbiota-derived metabolite generated from ellagic acid-rich dietary sources (such as pomegranate, berries, and walnuts), demonstrated potential antiproliferative properties through modulation of mitochondrial function, promotion of apoptosis, cell-cycle arrest, and anti-inflammatory activities (8, 10). UB is generated in the gut through microbial metabolism of dietary ellagitannins, a process carried out primarily by *Gordonibacter urolithinfaciens* and *Gordonibacter pamelaeae* (family *Eggerthellaceae*), with *Ellagibacter isourolithinifaciens* also contributing to urolithin production (11, 12). The efficiency of UB production varies widely across individuals, reflecting differences in gut microbiota composition (13). Combining these metabolites with standard chemotherapy may offer superior therapeutic advantages, particularly in treatment-resistant cancers such as lymphoma, given their complementary mechanisms of action.

Previous studies have established that N and UB independently exert potent antiproliferative and pro-apoptotic activities across various cancer cell types, including breast, colorectal, and oral squamous cell carcinoma (9, 10). Our recent research demonstrated synergistic interactions between these postbiotic metabolites, indicating antiproliferative activity and tumor-selective cytotoxicity (9, 14–17). Nonetheless, comprehensive evaluations involving multi-agent combinations, specifically N, UB, and Vinc, remain unexplored, particularly their combined potential against lymphoma cells. Recent studies demonstrate that postbiotics, including bacteriocins such as N and gut microbial metabolites like urolithins, exert anticancer effects through mechanisms involving apoptosis induction, cell cycle arrest, and modulation of the tumor microenvironment (18–20). Moreover, combinatorial approaches integrating natural products with standard chemotherapeutics are increasingly recognized as strategies to enhance efficacy and reduce toxicity (21).

Advancements in proteomic technologies, particularly label-free quantitative proteomics utilizing liquid chromatography-mass spectrometry (LC-MS/MS), have enabled sophisticated analyses of cellular responses to drug treatments. These approaches facilitate identifying and characterizing molecular pathways modulated by therapeutic combinations, revealing precise mechanistic insights into drug synergy (9, 16, 17, 22). Such in-depth molecular analyses can significantly guide therapeutic strategies and the clinical translation of promising drug combinations.

Therefore, this study aimed to systematically assess the antiproliferative and apoptotic synergy of N, UB, and Vinc combinations in human lymphoma cell lines (HKB-11 and Hs 313.T). We utilized *in vitro* assays, including Alamar Blue cytotoxicity assays, ROS measurements, and annexin V/7-AAD-based flow cytometry to quantify apoptosis. Additionally, comprehensive proteomic profiling via LC-MS/MS was conducted to elucidate the underlying molecular mechanisms driving the observed synergistic effects.

## 2 Materials and methods

### 2.1 Chemicals and drug preparation

N, UB, and Vinc were purchased from Sapphire Bioscience (Redfern, NSW, Australia), and doxorubicin (Dox) was purchased from Sigma Aldrich (Castle Hill, NSW, Australia). Furthermore, all reagents were prepared according to the standard methods and protocols provided with the assay kits.

### 2.2 Cell culture

Hs 313.T (ATCC CRL-7235), HKB-11 (ATCC CRL-12568; human kidney/B cell Hybrid) and HS-5 (ATCC CRL-3611) were purchased from the American Type Culture Collection (ATCC, Manassas, Virginia, United States). Hs 313.T lymphoma cells were grown in the ATCC-formulated Dulbecco’s Modified Eagle’s Medium (DMEM; ATCC 30-2002), comprised of 4.5 g/L glucose, L-glutamine, and sodium pyruvate supplemented with 10% fetal bovine serum (FBS; Bio-Strategy PTY Campbellfield, VIC, Australia), and supplemented with 1% penicillin and streptomycin (Sigma Aldrich, Castle Hill, NSW, Australia). The HKB-11 cells were grown in the ATCC-formulated DMEM: F12 (1:1 mixture of DMEM and Ham’s F-12) supplemented with 10% FBS (Bio-Strategy PTY Campbellfield, VIC, Australia) and supplemented with 1% penicillin and streptomycin (Sigma Aldrich, Castle Hill, NSW, Australia). HS-5 normal cells were grown in the ATCC-formulated DMEM (ATCC 30-2002), comprised of 4.5 g/L glucose, L-glutamine, and sodium pyruvate, supplemented with 10% FBS (Bio-Strategy PTY Campbellfield, VIC, Australia), and supplemented with 1% penicillin and streptomycin (Sigma Aldrich, Castle Hill, NSW, Australia). These cells were maintained at 37°C in a 5% controlled CO_2_ atmosphere, and cell maintenance was performed every 48–72 h, which is the time necessary for cells to achieve confluent monolayers.

### 2.3 Cell viability assays

The cell viability of the HKB-11, Hs313.T and HS-5 cells after treatment with different concentrations of N and UB, and the chemotherapy Vinc, were determined using the Alamar Blue assay as per the method described earlier (14–16, 22). Briefly, 100 μL of cells were cultured in 96-well plates at a 3 × 10^5^ cells/mL seeding density. After 24 h, the cells were treated with N: UB (3500 = 3200: 300 μM), Vinc (0.94, 0.63 and 0.31 μM), N: UB: Vinc at 2450.94 (2240: 210: 0.94 μM), 2800.63 (2560: 240: 0.63 μM) and 3150.31 (2880: 270: 0.31 μM), using a 1:2 serial dilution across a 6-point dose-response curve, followed by a 72-h incubation. A positive control using doxorubicin was prepared at a concentration of 4 μM, and an untreated control with 0.1% DMSO was added to every plate. At the end of the incubation period, the culture media were removed, and 100 μL of a 0.1 mg/mL Alamar Blue solution (resazurin, prepared as a stock solution at 1 mg/mL in freshly made PBS, followed by a 1:10 dilution with serum-free media) was added to each well and was further incubated for another 2.5 h. The fluorescence levels were assessed using a microplate spectrophotometer (BMG CLARIOstar, Mornington, VIC, Australia) with an excitation wavelength of 555 nm and emission measurement at 595 nm. The compounds were tested in triplicate, with the untreated control taken as 100% cell viability.

### 2.4 Synergy analysis

In our previous study, we combined N and UB at nine different ratios and found that the 4:6 ratio was the most potent combination against HKB-11 lymphoma cells (15). In this study, different doses of the 4:6 combination were further combined with different doses of Vinc. This study used the CI analysis model to show the interaction between N and UB. CompuSyn version 2.0 (Biosoft, CA, United States) was used for our calculations. Moreover, this software calculates CI based on the median-effect equation from the mass action law (16). The current study employed the CI model to investigate the nine pairwise postbiotic combinations with Vinc, using a six-point dose-response curve. The CI model quantifies the potential interactions between drug-drug combinations into three categories: (a) synergistic effect: CI value <1, (b) additive effect: CI = 1, and (c) antagonistic effect: CI value >1. The N: UB: Vinc combination at 2450.94 (2240: 210: 0.94 μM), 2800.63 (2560: 240: 0.63 μM), and 3150.31 (2880: 270: 0.31 μM) was further studied on molecular assays.

### 2.5 Analysis of ROS production

The effect of N: UB: Vinc and their combinations on the oxidative stress of the lymphoma cells was studied as per the protocol using the H2DCFDA (2′,7′-dichlorofluorescein diacetate) cellular ROS Detection Assay Kit (#ab113851; Abcam, Melbourne, VIC, Australia) (9, 22). Briefly, HKB-11 lymphoma cells (2.5 × 10^5^ cells/mL) were cultured in a 96-well plate, allowed to adhere overnight, and treated with 20 μM H2DCFDA for 45 min to assess ROS levels. The dye solution was removed, and cells were washed with 1× buffer. Next, the cells were treated with N: UB at 3500 μM (3200: 300 μM), 1750 μM (1600:150 μM), N: UB: Vinc at 2450.94 (2240: 210: 0.94 μM), 1225.47 μM (1120: 105: 0.47 μM), 2800.63 (2560: 240: 0.63), 1400.31 μM (1280: 120: 0.32 μM), 3150.31 (2880: 270: 0.31 μM) and 1575.16 μM (1440: 135: 0.16 μM), Dox (4 μM), and tert-Butyl hydroperoxide (TBHP) (150 μM), and then incubated at 37°C for 4 h. Finally, the plate was immediately read at Ex/Em = 485/535 nm using a microplate spectrophotometer (BMG CLARIOstar, VIC, Australia). The fold-change in ROS production was determined relative to the untreated control (cells treated with the supplement buffer according to the manufacturer’s protocol).

### 2.6 Flow cytometry analyses of the apoptotic profiles

The impact of N: UB, Vinc and their combinations on the apoptosis profiles of the HKB-11 lymphoma cells after 24 h treatment was studied using an annexin V and 7-AAD-based kit (#ab214663, Abcam, Melbourne, VIC, Australia) (9, 14, 15, 22). The HKB-11 cells were cultured in T75 cell culture flasks with an initial density of 1 × 10^6^ cells per 10 mL at 37 °C in the presence of 5% CO_2_ for 24 h. The following day, the cell culture media was removed from each flask and replaced with fresh FBS-containing media. The cultured flasks were then treated with the highest concentration of N: UB at 3500 (3200: 300 μM) as per our previous study, but a new set was conducted to re-evaluate the previous study outcomes in the current study, and the chemotherapy drug Vinc at three doses (0.94, 0.63 and 0.31 μM), and N: UB: Vinc at three doses, along with the positive control Dox (4 μM). FBS-containing medium was used as the untreated control. The flasks were then incubated at 37°C with 5% CO_2_ for 24 h. Then, the cell culture media from each flask were collected. Subsequently, trypsin (0.25% w/v) was added to the flasks and incubated for 4 min at 37°C. The trypsin reaction was neutralized with an equal volume of 10% FBS serum-containing media, and the cells were combined with the previously collected media. The cell pellets were obtained by centrifuging at 500 × g for 5 min at room temperature (RT). This procedure was repeated by suspending the cell pellets in 1 mL of PBS each time. The collected cell pellets from each treatment were immediately suspended in 500 μL of 1× binding buffer and gently mixed by pipetting. Annexin V-CF Blue (5 μL) and 7-AAD (5 μL) staining solutions were added to 100 μL of the cell suspension. The stained cells were incubated for 15 min in the dark at RT, after which 400 μL of a 1x assay buffer was added to each cell suspension. Subsequently, the cells were examined using a flow cytometer (Novocyte 3,000, ACEA Biosciences Inc., CA, United States), and data analysis and processing were performed using NovoExpress software (version 1.5.0, ACEA Biosciences Inc., CA, United States). In the initial step, the cells were gated on forward and side scatter modes to exclude cell aggregates and debris near the origin. The cells were then gated on dot plots, where Annexin V-CF in Pacific Blue was plotted against 7-AAD fluorescence in PerCP. Quadrants were positioned relative to the untreated control, indicating live cells (+Annexin V and − 7-AAD) appearing in the lower-left quadrant, early apoptotic cells (+Annexin V and − 7-AAD) in the lower-right quadrant, late apoptotic cells (+Annexin V and + 7-AAD) in the upper-right quadrant, and necrotic cells (−Annexin V and + 7-AAD) in the upper-left quadrant. For statistical analyses and visualization, the percentage data of cells in each quadrant after different treatments (n = 6) were exported to GraphPad Prism software (version 9.0, San Diego, CA, United States).

### 2.7 Liquid chromatography-mass spectrometry -driven bottom-up proteomics analysis

#### 2.7.1 Cell culture, treatment, and protein extraction

The HKB-11 lymphoma cells were placed in 6-well plates at a 3 × 10^6^ cells/well density and incubated overnight at 37°C in 5% CO_2_. After removing the media, it was replaced with fresh DMEM/F-12 medium supplemented with 10% FBS, and the cultured flasks were treated with specific doses of N: UB, Vinc and their combinations. Treatments were done in triplicate and incubated for 24 h under the same conditions. Following incubation, each flask of cells was subjected to a 0.25% w/v trypsin treatment for 4 min at 37°C, and the cell culture medium was collected. Additionally, an equal volume of DMEM F-12 medium (containing 10% FBS) was added before mixing with the previously collected media to neutralize the trypsin. The cells were spun in a centrifuge at 500 × g for 5 min at RT. The cell pellets were washed twice with ice-cold PBS and spun again at 500× g for 5 min. These cell pellets were then suspended in a lysis buffer that included 1 μL of universal nuclease (Easypep Mini Kit) and supplemented with Halt™ Protease and Phosphatase Inhibitor Cocktail in a 1:100 ratio (Thermo Fisher Scientific, Sydney, NSW, Australia). The cells were gently pipetted 10–15 times to reduce the sample’s viscosity and then placed on ice for 20 min. The lysate was centrifuged at 14,000 rpm for 20 min at 4°C, and the resulting liquid was collected.

#### 2.7.2 Protein quantification

The Pierce™ Rapid Gold BCA Protein Assay Kit (#A53226, Thermo Fisher Scientific, Sydney, NSW, Australia) was used to determine the protein concentration of the cell lysate in triplicate, using a bovine serum albumin (BSA) standard, following the manufacturer’s protocol (9, 14, 15, 17). In brief, 1 μL of each sample replicate was diluted 1:20 in Milli-Q water, along with 20 μL of each standard, and then placed in a 96-well plate with 200 μL of working reagent in each well. Samples were diluted to a concentration within the 20–2,000 μg/mL working range. The plate was thoroughly mixed on a plate shaker for 30 s, incubated at RT for 5 min, and then the absorbance was measured within 20 min at 480 nm using a microplate spectrophotometer (BMG CLARIOstar, Melbourne, VIC, Australia). The blank absorbance was subtracted from all other readings of standards and samples, and the sample concentration was determined using the established BSA standard calibration curve. The samples were then stored at −80°C for further analysis.

#### 2.7.3 Peptides preparation and clean-up

The protein samples (100 μg) were subjected to chemical and enzymatic sample processing using the EasyPep™ Mini MS Sample Prep Kit following the manufacturer’s instructions (Thermo Fisher Scientific, Sydney, NSW, Australia) and as reported in the literature (9, 17, 23). Briefly, the sample volume was adjusted to 100 μL using a lysis buffer in a microcentrifuge tube. Subsequently, the reduction and alkylation solutions (50 μL each) were introduced, gently mixed, and incubated at 95°C with a heat block for 10 min. The samples were allowed to cool to RT, after which 50 μL of the reconstituted trypsin/lys-C protease mixture was added to each sample and incubated with shaking at 37°C for 3 h. Following incubation, 50 μL of a digestion stop solution was gently mixed into the samples. Peptide clean-up columns were used to remove both hydrophilic and hydrophobic impurities. The resulting clean peptide samples were dehydrated using a vacuum centrifuge and reconstituted in 100 μL of a 0.1% formic acid solution in water for LC–MS analysis. Subsequently, these samples were carefully transferred to maximum recovery sample vials (Waters Corp., Milford, MA, United States).

#### 2.7.4 Label-free quantitative proteomics using micro-high-performance liquid chromatography coupled with quadruple time-of-flight mass spectrometry

##### 2.7.4.1 Liquid chromatography and mass spectrometry setup

Label-free, bottom-up proteomic quantification was performed using a micro-high-performance liquid chromatography system (Waters M-Class) coupled with a SCIEX™ TripleTOF^®^ 6600 quadrupole time-of-flight mass spectrometer, operated in positive electrospray ionization mode (ESI+). A total of 4 µg of tryptic peptide digest was injected onto a nanoEase M/Z HSS T3 column (1.8 µm, 300 µm × 150 mm; Waters, 186009249) with an in-line Zorbax 300SB-C18 guard column (5 µm, 5 × 0.3 mm; Agilent Technologies, USA). The column temperature was maintained at 40°C. Mobile phase A consisted of 98% water and 2% acetonitrile, and mobile phase B consisted of acetonitrile with 0.1% formic acid. The system operated at a flow rate of 5 µL/min, with loading and column washing steps conducted at 7 µL/min. The LC gradient was as follows: 2–10% B over 1.66 min at 7 µL/min, 10–25% B from 1.67 to 21.67 min at 5 µL/min, followed by a sharp increase to 95% B from 23.33 to 24.67 min, held for 2 min, and re-equilibrated at 2% B for 9 min at 7 µL/min.

##### 2.7.4.2 Mass spectrometry acquisition parameters

The mass spectrometer had a DuoSpray™ ion source and a 25 μm internal diameter electrode. Data were acquired using the Analyst 1.8.1 software suite and associated LC control drivers. Key ion source parameters were: GS1 = 25, GS2 = 15, curtain gas = 20, ion spray voltage floating = 5500 V, and ion source temperature = 150 °C. The acquisition employed the SWATH™ data-independent acquisition (DIA) strategy, comprising an MS1 survey scan (m/z 350–1250, 50 ms accumulation) followed by 40 variable-width MS2 windows (m/z 400–1250), each with a 35 ms accumulation time, covering the full precursor m/z range. MS2 spectra were acquired in high-resolution mode across m/z 100–2000, with an overall cycle time of approximately 1.5 s.

##### 2.7.4.3 Mass calibration and library generation

PepCalMix calibrant (SCIEX, P/N 5045759; 10 fmol/µL), diluted 1:100 in 5% acetic acid and 2% acetonitrile, was injected every 12 samples to ensure mass accuracy. Six pooled quality control (QC) samples were used to construct a DIA-only spectral library using a gas-phase fractionation approach (24), covering m/z segments: 400–500, 500–600, 600–700, 700–800, 800–900, and 900–1000. The precursor isolation window was set to 5 m/z, with a collision energy spread of 5 eV, except for m/z 700–990 (8 eV) and 990–1000 (10 eV). Each DIA segment cycle time was 2.14 s, incorporating low- and high-energy scans with 40 ms MS2 accumulation.

##### 2.7.4.4 Data processing and statistical analysis

Data were processed using Spectronaut v19.5, which implemented the DirectDIA+ workflow with the Biognosys Standard (BGS) analysis framework. The canonical human reference proteome (UniProt, released 24 January 2024; 17,179 entries) was used as the reference database. Searches were conducted using Pulsar, with enzymatic specificity for trypsin/P and LysC/P, allowing up to 2 missed cleavages. Peptide lengths were restricted to 7–52 amino acids. Carbamidomethylation (C) was set as a fixed modification, while variable modifications included protein N-terminal acetylation, methionine oxidation, and methylation and demethylation. A maximum of five variable modifications per peptide was permitted. Peptide-spectrum matches (PSMs), peptides, and protein groups were filtered at a % false discovery rate (FDR) of 1%. Label-free quantification (LFQ) was performed automatically using MS2 area integration with default normalization strategies. Protein inference employed the IDPicker algorithm.

Ingenuity Pathway Analysis (IPA, Qiagen) was implemented for pathway enrichment analyses using differentially expressed proteins (DEPs) at absolute Log_2_FC (0.58 and Q 0.05) to identify canonical pathways responsible for the antiproliferative mechanisms of mono-treatment versus control and the synergistic effects of combinations versus mono-treatments against HKB-11 lymphoma cells.

### 2.8 Statistical analysis

Data were collected and managed using MS Office Excel and GraphPad Prism for statistical analyses and visualization. Data collection and analyses were carried out in triplicate, and the outcomes were presented as the mean ± standard deviation. Statistical significance between the mean values was determined at p < 0.05 employing a two-way ANOVA. Tukey and Dunnett’s tests were utilized within the GraphPad Prism software to perform nonlinear regression and multiple comparisons. Furthermore, GraphPad Prism software computed the IC_50_ value (representing the drug concentration required to achieve a 50% cell growth inhibition). The experimental groups in the proteomics study were compared statistically using unpaired t-tests, assuming equal variances. Candidate proteins were selected based on an absolute log_2_ fold change ≥ 0.58 and a Q-value ≤ 0.05. Enrichment analysis was subsequently conducted using Ingenuity Pathway Analysis (IPA), applying a significance threshold of adjusted Q ≤ 0.05 to identify functionally enriched pathways, focusing on those with an absolute z-score of ≥1.

## 3 Results and discussion

### 3.1 Antiproliferative activity of the N, UB and Vinc combinations against the HKB-11 and Hs 313.T lymphoma cells, and their synergistic effects against HKB-11

**Table 1** demonstrates that combinations of N, UB, and Vinc consistently produced CI values below 1 across all tested dose levels in HKB-11 lymphoma cells, signifying synergistic interactions (25). The concentrations for the N:UB: Vinc combinations (e.g., 2240:210:0.94 µM) were derived from previous optimization of N:UB at a 4:6 ratio, which showed the strongest synergy against HKB-11 cells (CI = 0.09 at IC95) (15). Vinc was introduced at sub-micromolar doses to preserve efficacy while minimizing off-target toxicity. Notably, Vinc doses used fall within the lower range of therapeutic plasma levels (27), while N and UB doses were selected based on *in vitro* optimization (14, 15), in which the N: UB 4:6 (3200: 300 μM) combination demonstrated highly synergistic effects (CI = 0.09 at IC_95_) against HKB-11 cells, with selectivity for malignant lymphoma cells over normal cells. This prior observation formed the basis for using N: UB 4:6 (3200: 300 μM) as the foundation for combining it with Vinc in this study, underscoring a strategic, data-driven approach to combination therapy by reducing the chemotherapy concentration while keeping the same effect or increasing it.

**Table 1:** Drug interaction analysis in HKB-11 lymphoma cells of nisin (N), urolithin B (UB), and vincristine (Vinc) combinations, expressed as the combination index (CI).

Concentration (μM) N: UB: Vinc	Combination index (CI) at:			
	IC_50_	IC_75_	IC_90_	IC_95_
320: 30: 2.81	**0.38**	**0.77**	1.73	3.12
640: 60: 2.5	**0.33**	**0.51**	**0.85**	1.29
960: 90: 2.2	**0.44**	**0.48**	**0.6**	**0.72**
1280: 120: 1.9	**0.42**	**0.44**	**0.52**	**0.6**
1600: 150: 1.6	**0.36**	**0.37**	**0.42**	**0.47**
1920: 180: 1.25	**0.31**	**0.35**	**0.42**	**0.5**
2240: 210: 0.94	**0.36**	**0.41**	**0.51**	**0.6**
2560: 240: 0.63	**0.42**	**0.47**	**0.54**	**0.61**
2880: 270: 0.31	**0.39**	**0.41**	**0.43**	**0.45**

Combination Index (CI). Inhibitory concentration (IC) at _50_, _75_, _90_ and _95_. The bold numbers (CI values < 1) indicate synergistic interactions between N, UB and Vinc, where the top two synergistic interactions at each IC were underlined.

These data demonstrated that introducing Vinc into an already effective natural metabolite regimen maintains, and in some instances enhances, the synergistic inhibition of lymphoma cell growth. The combination of N, UB, and Vinc demonstrated dose-dependent efficacy and selectivity in inhibiting lymphoma cell growth while sparing normal stromal cells at lower doses (**Table 2**). Furthermore, as shown in **Table 2**, the triple combination caused near-complete inhibition of both HKB-11 and Hs 313.T lymphoma cells at higher doses. For instance, the combination at 2880: 270: 0.31 μM yielded 99.91 ± 0.27% inhibition in HKB-11 cells and 100.42 ± 1.38% in Hs 313.T, however, was also toxic to the normal stromal HS-5 cells (3.97 ± 1.60% viability). At a mid-range concentration of 560: 52.5: 0.23 µM, the combination remained highly effective against lymphoma cells (98.55% inhibition in HKB-11 and 93.88% in Hs 313.T) while maintaining relatively moderate toxicity toward HS-5 cells (19.89% viability). This concentration reflects a favorable balance between anticancer efficacy and safety toward normal cells. At lower concentrations (e.g., 90: 8.4: 0.01 μM and below), the combination was more effective in inhibiting the Hs 313.T lymphoma cells (> 84%), reflecting a favorable therapeutic index. However, at these lower concentrations tested, the combination showed a marked reduction in cytotoxicity against HKB-11 lymphoma cells. For instance, at 90: 8.4: 0.01 µM and below, the HKB-11 cells were inhibited by 19-33%, while HS-5 cell viability increased to > 40%. The observed variation in toxicity between HKB-11 and Hs 313.T may be due to differences in apoptotic thresholds, drug metabolism, or efflux transporter expression (26).

**Table 2:** Cell growth inhibition (%) against HKB-11 and Hs 313.T lymphoma cell lines and cell viability (%) of HS-5 normal stromal cell line at different concentrations of nisin (N), urolithin B (UB) and vincristine (Vinc) combinations.

Concentration (μM) N: UB: Vinc	Cell growth inhibition (%)		Cell viability (%)
	HKB-11	Hs 313.T	HS-5
2880: 270: 0.31	99.91 ± 0.27 ^a^	100.42 ± 1.38 ^a^	3.97 ± 1.60
2560: 240: 0.63	99.51 ± 0.08 ^a^	100.12 ± 1.03 ^a^	4.63 ± 0.67
2240: 210: 0.94	98.38 ± 1.23 ^a^	100.16 ± 1.46 ^a^	5.33 ± 0.69
1440: 135: 0.16	99.14 ± 0.19 ^a^	98.00 ± 1.05 ^a^	9.67 ± 0.17
1280: 120: 0.31	98.69 ± 0.35 ^a^	98.32 ± 0.96 ^a^	8.97 ± 0.19
1120:105: 0.47	99.20 ± 0.50 ^a^	97.52 ± 2.10 ^a^	8.81 ± 0.44
720: 67.5: 0.08	98.81 ± 0.15 ^a^	96.41 ± 1.03 ^a^	16.19 ± 0.53
640: 60: 0.16	97.96 ± 0.89 ^a^	96.94 ± 1.41 ^a^	17.80 ± 1.73
560: 52.5: 0.23	98.55 ± 0.77 ^a^	93.88 ± 1.72 ^a^	19.89 ± 0.46
360: 33.8: 0.04	91.73 ± 2.83 ^a^	94.81 ± 3.34 ^a^	32.23 ± 0.99
320: 30: 0.08	81.54 ± 4.52 ^a^	94.67 ± 1.99 ^b^	29.43 ± 1.33
280: 26.3: 0.12	82.03 ± 5.56 ^a^	91.36 ± 1.32 ^b^	30.34 ± 0.30
180: 16.9: 0.02	53.48 ± 5.94 ^a^	89.01 ± 2.75 ^b^	38.64 ± 1.10
160: 15: 0.04	41.82 ± 3.63 ^a^	91.18 ± 2.94 ^b^	34.93 ± 0.54
140: 13.1: 0.06	47.28 ± 4.03 ^a^	91.22 ± 3.06 ^b^	34.35 ± 1.30
90: 8.4: 0.01	19.13 ± 2.68 ^a^	84.43 ± 0.79 ^b^	40.90 ± 1.22
80: 7.5: 0.02	27.89 ± 2.69 ^a^	86.00 ± 1.07 ^b^	41.35 ± 0.50
70: 6.6: 0.03	33.86 ± 0.89 ^a^	86.08 ± 1.22 ^b^	42.09 ± 1.21

Data are presented as mean ± standard deviation (SD). ^a,b^ values in the same row not having the same superscript letter are significantly different (p < 0.05) at the same concentration.

These data suggest that while the combination loses efficacy at lower doses against HKB-11 cells, it retains its activity against Hs 313.T lymphoma cells and maintains a favorable toxicity profile toward HS-5 normal cells. The combination doses (e.g., 560:52.5:0.23 µM) were optimized for *in vitro* synergy. N and UB exceed expected systemic concentrations, highlighting the need for advanced delivery systems to improve physiological relevance. Overall, the combination exhibited strong and selective cytotoxicity against lymphoma cells, with the 560: 52.5: 0.23 µM dose emerging as a promising therapeutic window. Our findings align with the clinical principle that combination therapy can achieve superior efficacy compared to monotherapy. The CHOP regimen exemplifies this paradigm, achieving durable remissions in non-Hodgkin lymphoma, but also causing significant toxicities, particularly from Vinc and doxorubicin (28, 29). By integrating postbiotics with Vinc, our data suggested a vinc-sparing approach that could complement, rather than replace, established chemotherapy regimens. Such strategies may enhance tumor inhibition while potentially mitigating dose-limiting toxicities, warranting further *in vivo* testing and exploration alongside standard regimens. Compared with Vinc, the N:UB: Vinc triple combination achieved stronger lymphoma cell inhibition in the ALAMAR blue assay, while requiring substantially lower Vinc exposure and conferring relative sparing of stromal cells. Benchmarking against our previously reported N: UB (4:6) combination (14). The addition of Vinc further enhanced efficacy and extended the therapeutic window. Although the triple combination was not directly compared with CHOP or other clinical regimens in this study, these findings suggest that combining postbiotics with Vinc offers a translationally relevant strategy for lymphoma treatment.

Also, lower concentrations may still offer value in clinical scenarios prioritizing reduced systemic toxicity. Dose-response behavior is crucial for treatment regimens seeking to balance efficacy with safety, particularly in diseases like lymphoma, which often require long-term management.

Vinc, while effective, is known for its dose-limiting neurotoxicity and systemic side effects (3). By leveraging synergistic interactions with bioactive postbiotics such as N and UB, it may be possible to lower the necessary dose of Vinc without sacrificing therapeutic potency much. Although Vinc is well known to cause dose-limiting toxicities in patients, including peripheral neuropathy, myelosuppression, and gastrointestinal toxicity, even at therapeutic plasma levels (0.1–1 µM) (5, 30). Therefore, the ability to achieve comparable efficacy at lower Vinc doses in the triple combination represents a potential clinical advantage. Nonetheless, the stromal toxicity observed here warrants further validation in primary mesenchymal stromal cells and animal models to determine whether this effect is clinically acceptable.

Previously, we reported that the N: UB (4:6) combination at 437.5 μM significantly inhibited Hs 313.T growth (76.06 ± 4.43%) while sparing stromal HS-5 cells (66.37 ± 11.53%) (15). In combination regimens, Vinc can dominate the cytotoxic profile, especially when paired with non-toxic or moderately toxic agents such as N or UB. Therefore, the reduced viability of HS-5 cells in the lower micromolar range likely reflects Vinc-driven cytotoxicity rather than an inherent effect of the postbiotics. The HS-5 viability values (19-33%) observed *in vitro* indicate measurable stromal toxicity but should not be interpreted as directly predictive of clinical tolerability. Given the limitations of immortalized HS-5 cells, further studies in primary stromal cells and *in vivo* models will be required to establish whether this protective trend is clinically meaningful.

The overarching goal of this study was to identify postbiotic and chemotherapy combinations that maintain or enhance antiproliferative activity while reducing the toxic burden associated with standard chemotherapy. This concept is supported by studies showing that combination regimens can enhance efficacy and reduce side effects through mechanisms such as pathway convergence and metabolic targeting (6, 7).

### 3.2 ROS production after treatment with different concentrations of N: UB and Vinc in the HKB-11 lymphoma cells

**Figure 1:**
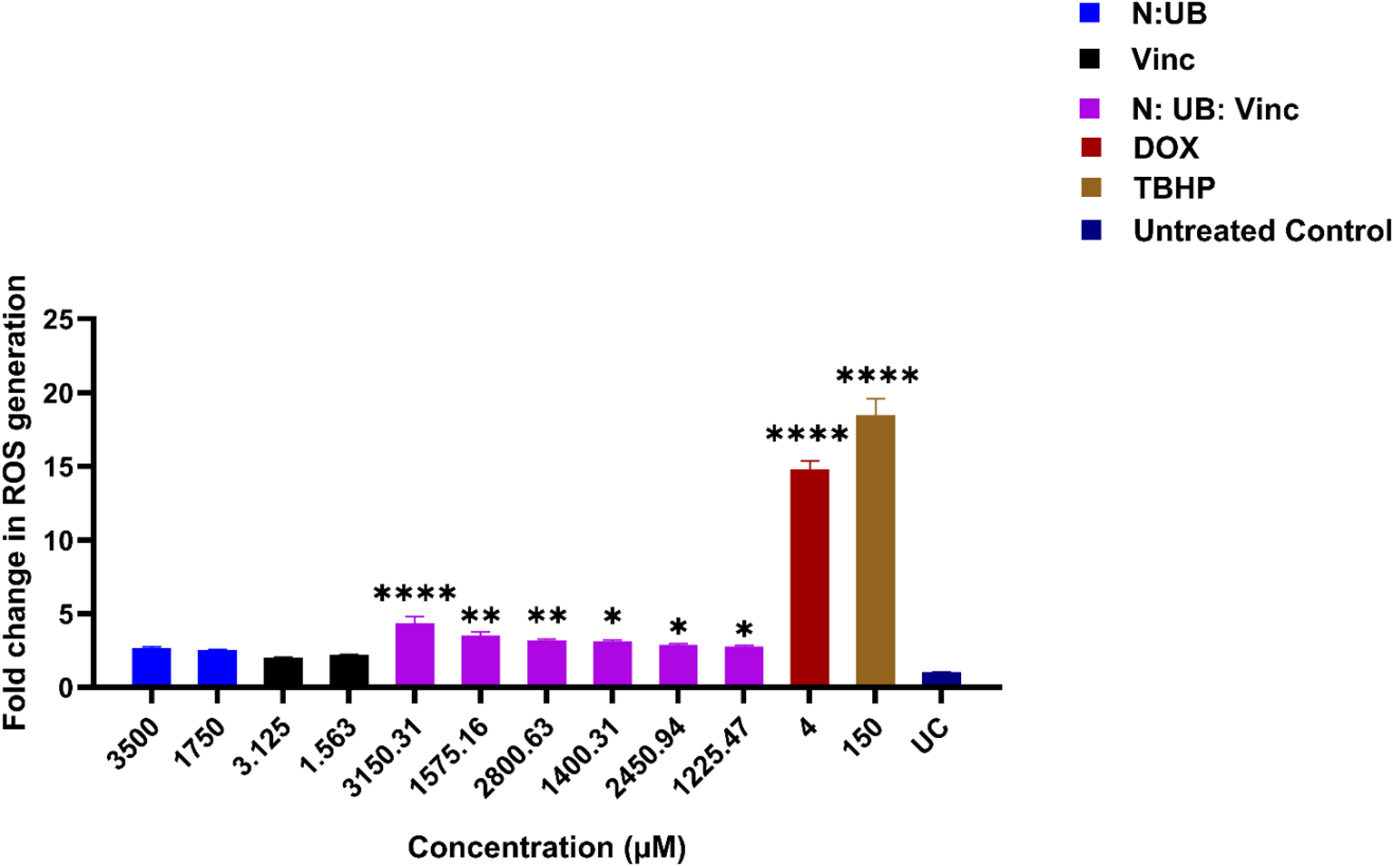
The effect of two different concentrations of the combination N: UB at 3500 (3200: 300 μM) and 1750 μM (1600: 150 μM), Vinc at 3.125 and 1.563 μM, N: UB: Vinc at 3150.31 (2880: 270: 0.31 μM) and 1575.16 μM (1440: 135: 0.16 μM), 2800.63 (2560: 240: 0.63), 1400.31 μM (1280: 120: 0.32 μM), 2450.94 (2240: 210: 0.94 μM), 1225.47 μM (1120: 105: 0.47 μM), on the production of ROS in the HKB-11 lymphoma cell line. For comparative purposes, Dox (4 μM) and tert-Butyl hydroperoxide (TBHP; 150 μM) were included. The values are expressed as mean ± SD. * indicates p <0.05; ** indicates p ≤ 0.01; **** indicates p ≤ 0.0001 compared to untreated control.

ROS are critically involved in cancer biology. While moderate ROS levels support tumor growth and survival, excessive accumulation can lead to oxidative damage, mitochondrial dysfunction, and apoptotic cell death (31–33). Therefore, regulating ROS presents a promising strategy for targeting cancer cells selectively. **Figure 1** illustrates the effects of various treatments of N: UB, Vinc and their combinations on ROS production in HKB-11 lymphoma cells.

As our previous study identified the N: UB 4:6 (3200:300 μM) combination as one of the most synergistic and selectively potent combinations against the tested lymphoma cells (14). It was evaluated again at 3500 μM (3200:300 μM) and 1750 μM (1600:150 μM) in this study for comparison. Consistent with prior observations, this combination induced a mild but insignificant increase in ROS levels compared to the untreated control (p > 0.05). This profile may be therapeutically meaningful, as it suggests the potential to induce anti-proliferative activity without overwhelming oxidative stress that could harm normal cells.

Vinc, a well-established chemotherapeutic, was evaluated at concentrations of 3.125 and 1.563 μM, with both concentrations showing no elevation in ROS production compared to the untreated control (p > 0.05). This lack of significance may imply that at these low doses, Vinc’s mechanism of cytotoxicity is likely independent of ROS elevation, consistent with its known action as a microtubule disruptor rather than a strong ROS inducer (34). However, combining N: UB with Vinc at various concentrations (3150.31- 1225.47 μM) resulted in a significant and dose-dependent increase in ROS levels, with the most substantial effect observed at the highest tested dose of 3150.31 μM (2880: 270: 0.31 μM). As expected, TBHP (150 μM) and Dox (4 μM), two positive controls known for their ROS-generating capacity (35, 36), induced significant ROS production in the HKB-11 lymphoma cells in this study.

The marked increase in ROS by the N: UB: Vinc combination suggested that Vinc may interact with N and UB to induce oxidative stress beyond the capacity of the N: UB combination alone. The triple combination likely overwhelmed the antioxidant defenses of the lymphoma cells, tipping them toward apoptosis or other forms of oxidative damage-mediated death (33, 37). Importantly, this effect is dose-dependent, allowing modulation of ROS levels based on therapeutic goals, such as milder treatment with selective cytotoxicity or aggressive ROS induction for advanced disease states.

Vinc disrupts microtubules to block cell division in rapidly growing cancer cells, but this same disruption affects neurons, which rely on microtubules for axonal transport. As a result, Vinc causes peripheral neuropathy, a significant dose-limiting side effect (5, 38). The approach of combining Vinc with postbiotics such as N and UB may have several benefits; it may allow for dose reduction of Vinc, thus minimizing its well-documented neurotoxicity and systemic side effects. Additionally, it leverages the synergistic mechanisms of action: N may initiate ROS production, Vinc enhances mitochondrial stress, and UB may fine-tune redox balance, enhancing tumor selectivity (6, 39). Third, ROS modulation can support subtype-specific therapy, especially in lymphomas with altered redox homeostasis or mitochondrial vulnerabilities (40).

### 3.3 Flow cytometric analysis of apoptotic profiles in HKB-11 cells treated with N: UB and N: UB: Vinc combinations

To build upon the previous study, which demonstrated the apoptotic potency of the N: UB combination (15), the current study employed flow cytometry to analyze the effects of adding Vinc to the N: UB combination against HKB-11 lymphoma cells. Cells were exposed for 24 h to several treatment groups: N: UB at 3500 µM (3200: 300 µM), Vinc alone at three concentrations (0.94 µM, 0.63 µM, 0.31 µM), and the combination of N: UB: Vinc at varying concentrations (2450.94 µM = 2240: 210: 0.94 µM; 2800.63 µM = 2560: 240: 0.63 µM; 3150.31 µM = 2880: 270: 0.31 µM). These groups were selected to evaluate the dose response of Vinc when combined with N and UB and to determine whether including this mitotic inhibitor could synergistically enhance apoptotic cell death.

**Figure 2:**
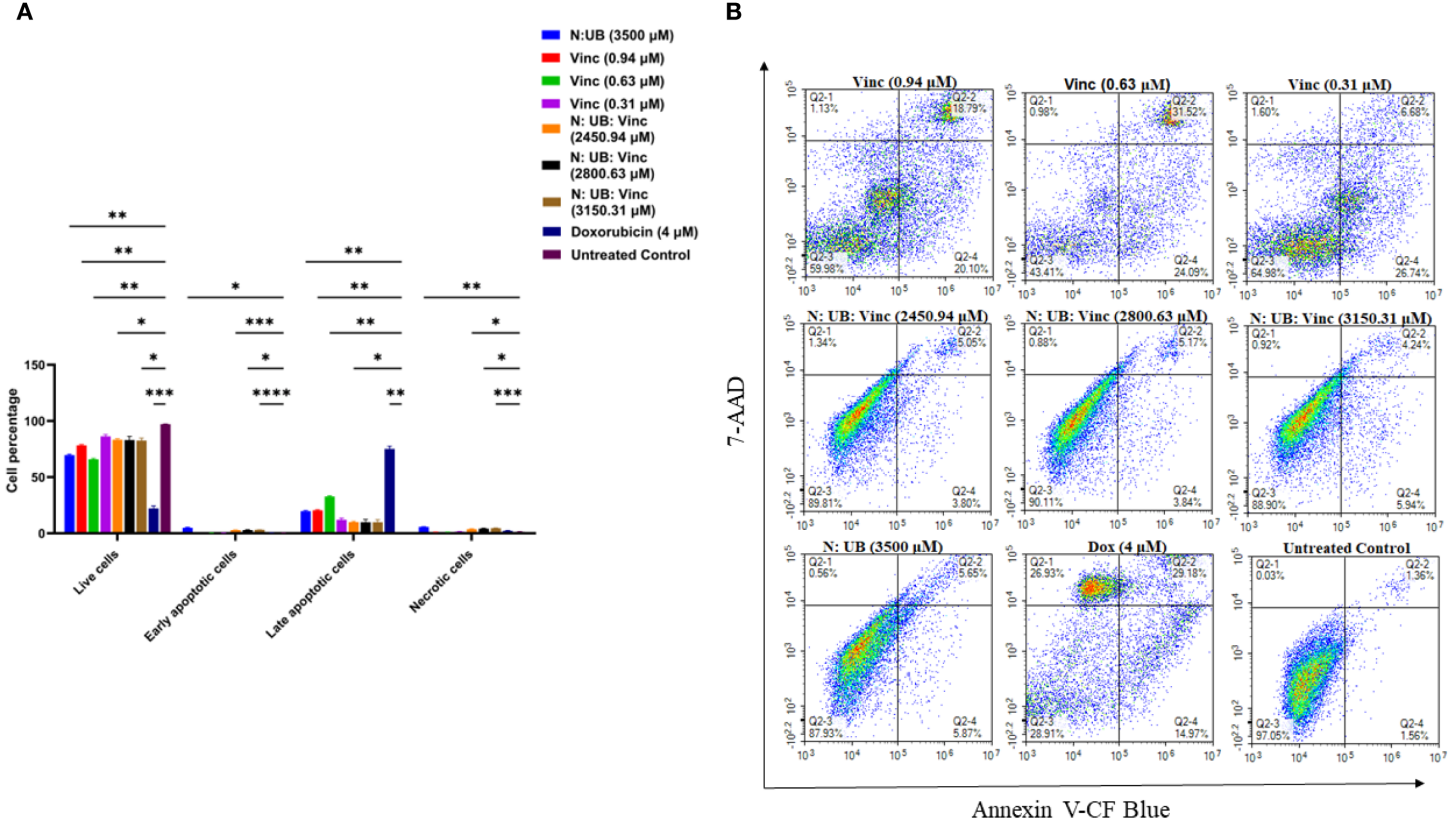
Flow cytometric assessment of the apoptotic profiles of the HKB-11 lymphoma cancer cells after 24 h of treatment. **(A)** The live, early apoptotic, late apoptotic, and necrotic cell percentages after 24 h treatment with N: UB at 3500 (3200: 300 μM), Vinc at (0.94, 0.63 and 0.31 μM), N: UB: Vinc at 2450.94 (2240: 210: 0.94 μM), N: UB: Vinc at 2800.63 (2560: 240: 0.63 μM) and N: UB: Vinc at 3150.31 (2880: 270: 0.31 μM) (*n* = 6), respectively. * indicates 0.01 < value of *p <*0.05; ** indicates *p* < 0.01; *** indicates *p* < 0.001; **** indicates *p* < 0.0001 compared to the untreated control. **(B)** The density plots of each drug treatment are represented, which are the most representative of the average data from the flow cytometric analyses. Q2–1 indicates necrotic cells, Q2–2 indicates late-stage apoptotic cells, Q2–3 indicates live cells, and Q2–4 indicates early-stage apoptotic cells.

As shown in **Figure 2A**, the N: UB combination at 3200: 300 µM demonstrated similar values compared to our last study, eliciting a marked increase in early and late apoptotic cells compared to untreated controls (p < 0.01). Moreover, this confirmed its baseline efficacy and validated the combination’s mechanistic synergy: N may induce ROS-mediated membrane disruption, while UB potentially targets mitochondrial oxidative phosphorylation to initiate intrinsic apoptosis (10, 14, 41). Importantly, necrotic cell death remained low, reinforcing the potential therapeutic selectivity of this combination. Also, when Vinc was administered alone at all tested concentrations 0.94, 0.63, and 0.31 µM compared to the untreated control (p < 0.01; p < 0.01; p < 0.01 and p < 0.05, respectively), it triggered a markedly higher apoptotic response than the N: UB and N: UB: Vinc combination. Interestingly, among all treatments, Vinc at 0.63 µM induced the highest total apoptosis, with early and late apoptosis outperforming Vinc at 0.94 µM as shown in **Figure 2B**. (42). This trend reflects Vinc’s well-established mechanism of action as a microtubule-disrupting agent (34). This phenomenon, where intermediate doses show maximal biological effect, has been reported for microtubule-targeting agents. At higher concentrations, vinca alkaloids can cause rapid microtubule depolymerisation, leading to mitotic slippage, necrosis, or other non-apoptotic death modes, rather than classical apoptosis (34). At lower doses, the drug may not reach the threshold needed to sustain mitotic arrest long enough to trigger full apoptotic cascades (43). Additionally, moderate microtubule perturbation can sustain mitotic checkpoint activation and caspase-dependent apoptosis without prematurely exiting mitosis (44). The observed superiority of Vinc compared to the combination in apoptosis induction in this study may also be time-dependent. Although vinc monotherapy induced high apoptosis at maximal doses, this effect was accompanied by pronounced stromal toxicity, reflecting vinc’s known dose-dependent toxicities, including peripheral neuropathy and myelosuppression (45, 46). The triple combination achieved equivalent lymphoma inhibition at substantially lower vinc concentrations while conferring relative sparing of normal stromal HS-5 cells. This vinc-sparing effect is clinically relevant, as vinc dose reductions are frequently required in lymphoma treatment due to toxicity (30). Thus, the therapeutic advantage of the triple combination lies in reducing vinc exposure while maintaining efficacy.

Postbiotics such as N and UB often exert their full anti-proliferative effects through pathways that require prolonged activation, including mitochondrial dysfunction, oxidative stress buildup, and modulation of gene expression over time (47, 48). These mechanisms are typically slower in action compared to microtubule-targeting agents, such as Vinc. As such, the shorter exposure of 24 h in the apoptosis assay may have underestimated the full potential of the N: UB combination. This discrepancy underscored the need to standardize drug exposure durations across assays. Failing to do so can misrepresent the comparative efficacy of time-sensitive agents, particularly postbiotics, whose mechanisms require extended engagement with cellular systems. It also highlighted the importance of integrating both short- and long-term cytotoxicity data to evaluate therapeutic synergy. Therefore, in this study, the Alamar Blue viability assay was performed after 72 h of exposure to understand the long-term effects, and flow cytometry and proteomics analyses were undertaken after 24 h to understand the short-term effects of the combination and its individual agents.

The untreated control and N: UB alone group (3500 µM) maintained high viability, with negligible apoptotic activity. Among the tested N: UB: Vinc combinations, 2880: 270: 0.31 μM significantly increased early cell populations (p < 0.0001) while maintaining a lower necrotic profile (p < 0.001) compared to the untreated control, as shown in **Figure 2A**. This combination also significantly (p < 0.0001) produced the highest apoptotic population among the N: UB: Vinc combination groups. Similar trends were observed in the other N: UB: Vinc doses (2800.63 µM and 2450.94 µM), with elevated late apoptosis but limited early response. Specifically, N likely contributes to ROS generation, membrane destabilization, and UB by activating the mitochondrial caspases. However, Vinc disrupts microtubule dynamics, leading to mitotic arrest and activation of the spindle assembly checkpoint (42). Together, these effects may trigger apoptosis via both intrinsic and extrinsic mechanisms. Interestingly, while the combinations altered the apoptotic signature toward a late apoptosis, they did not surpass Vinc alone at (0.63 µM) in total apoptotic induction. (42).

All tested combination groups and Vinc exhibited significantly lower necrotic population compared to the positive control Dox (p <0.001). While Dox induced strong apoptotic effects, it also triggered extensive necrosis, an outcome associated with pro-inflammatory responses and clinical side effects such as cardiotoxicity, mucositis, and systemic inflammation, as well as increased risks of exacerbated tissue damage and adverse systemic responses (49, 50).

Mechanistically, the efficacy of the N: UB: Vinc combinations may be attributable to integrating three apoptosis-inducing pathways. N was previously shown to initiate oxidative stress through ROS, UB to destabilize mitochondrial membrane potential and promote cytochrome c release, and Vinc to arrest mitosis, thus activating pro-apoptotic Bcl-2 family proteins and downstream caspases (51, 52). The ability to activate multiple apoptotic nodes simultaneously is particularly valuable in lymphoma, where drug resistance frequently arises through redundancy in survival signaling and inhibition of singular apoptotic pathways (42).

### 3.4 Proteomic characterization of HKB-11 cells treated with N: UB, Vinc, and their combination

**Figure 3:**
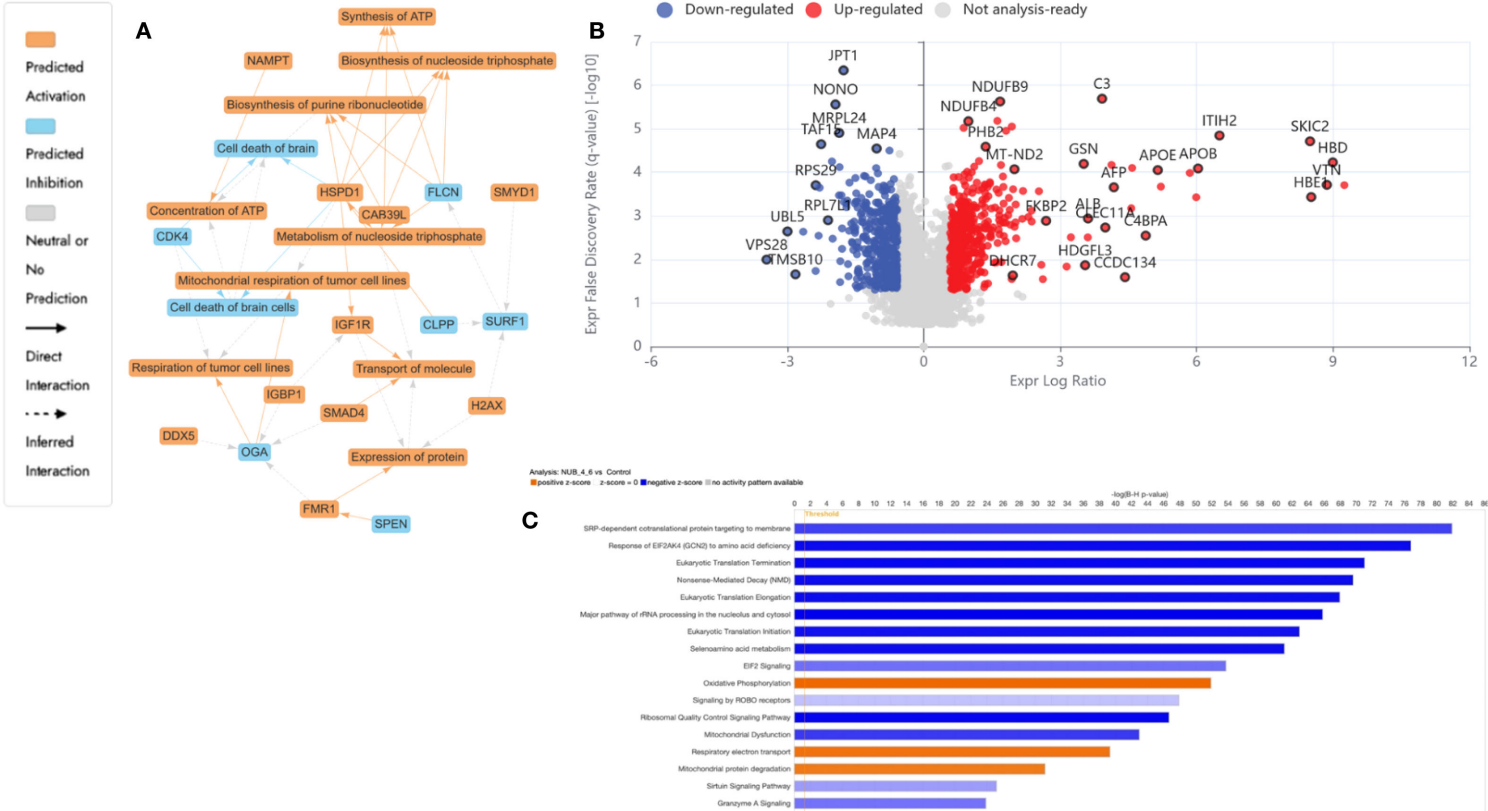
Proteomics analysis of nisin (N): urolithin B (UB) at 3500 µM (3200:300 µM)-treated HKB-11 lymphoma cells vs untreated control, showing **(A)** IPA graphical summary showing two biologically enriched themes. The significantly regulated proteins were identified (Q ≤ 0.05, log_2_ fold change ≥ 0.58). **(B)** Volcano plot of significantly regulated proteins (Absolute Log_2_ fold change ≥ 0.58 and Q ≤ 0.05). **(C)** Top IPA-enriched canonical pathways (|Z| >2 and Q <0.05).

**Figure 4:**
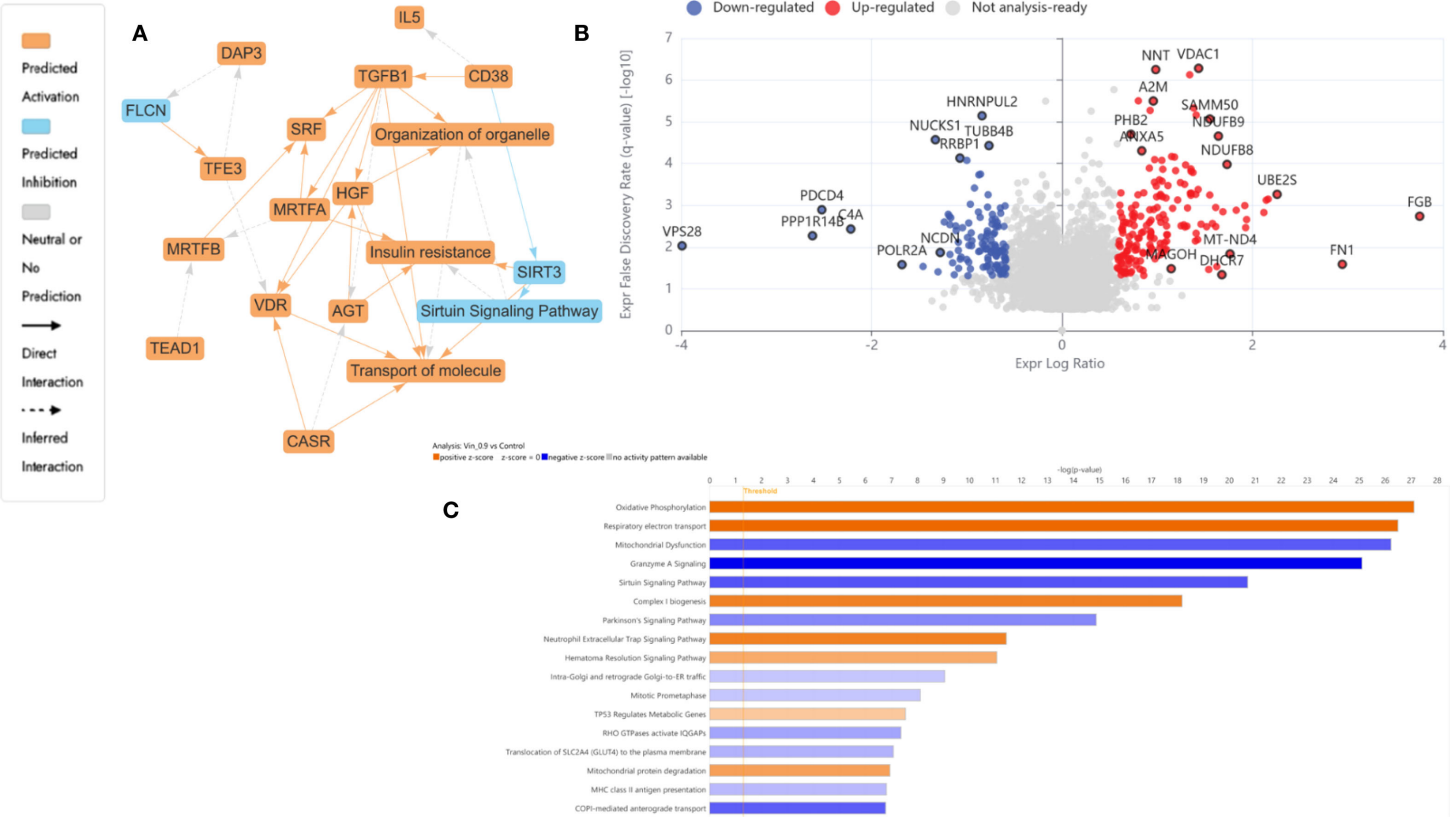
Proteomics analysis of Vinc at 0.94 µM treated HKB-11 lymphoma cells vs untreated control, showing **(A)** IPA graphical summary showing two biologically enriched themes. The significantly regulated proteins were identified (Q ≤ 0.05, log_2_ fold change ≥ 0.58). **(B)** Volcano plot of significantly regulated proteins (Absolute Log_2_ fold change ≥ 0.58 and Q ≤ 0.05). **(C)** Top IPA-enriched canonical pathways (|Z| >2 and Q <0.05).

**Figure 5:**
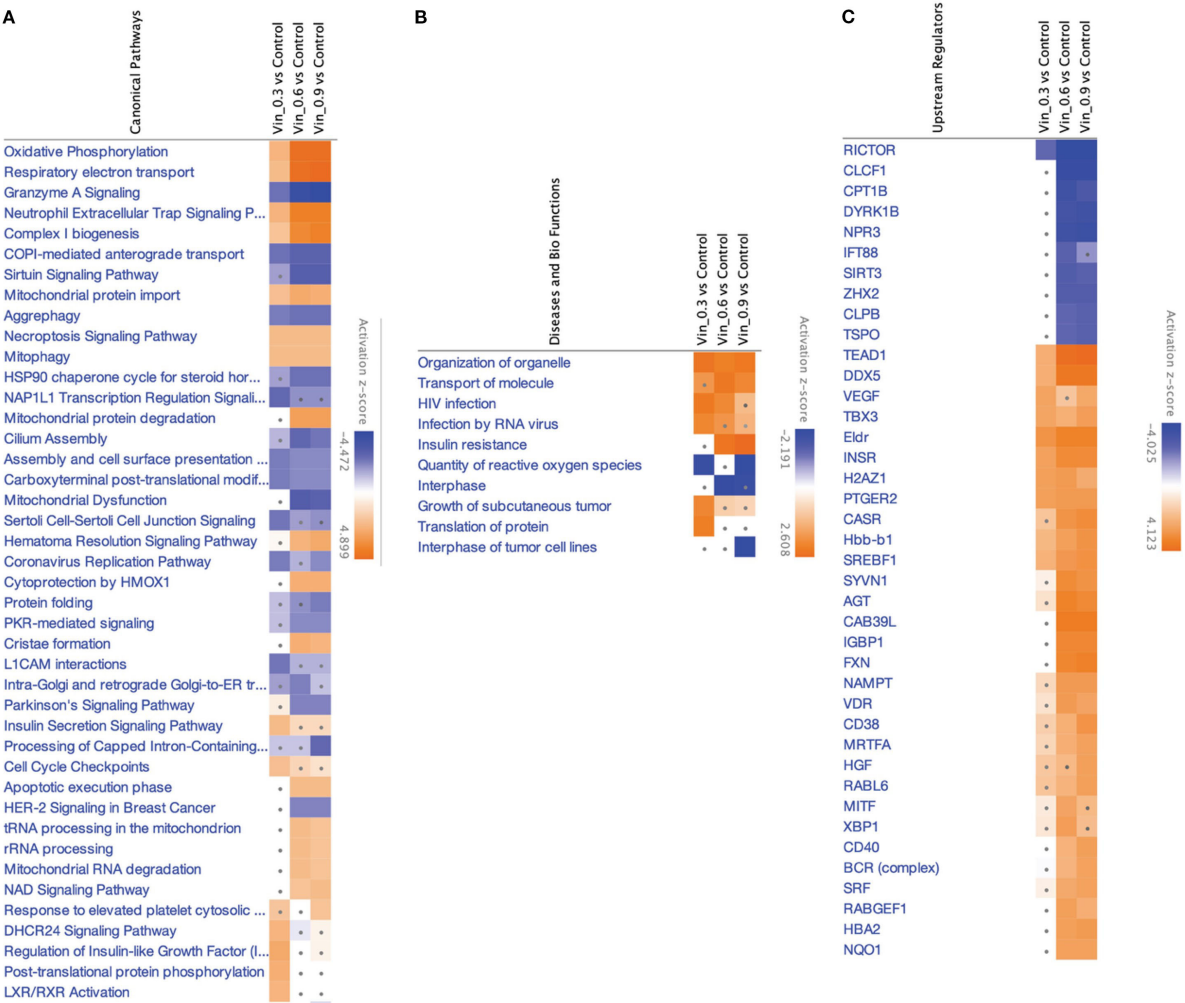
IPA analyses summary of different Vinc concentrations used for different synergistic combinations against HKB-11 cells at 2450.94, 2800.63 and 3150.31 μM using Differentially expressed proteins (DEPs) |Log_2_FC| ≥ 0.58, Q ≤ 0.05, showing **(A)** Significant canonical pathways (|Z-score|>1 and Q <0.01), **(B)** Enriched diseases and biofunctions (|Z-score|>2 and Q <0.01) and **(C)** Significant upstream regulators (|Z-score|>2 and Q <0.01). The dots indicate absolute z-scores of less than 2.

To unravel the molecular mechanisms underpinning the antiproliferative effects of N, UB, Vinc, and their combined administration on HKB-11 lymphoma cells, we employed label-free quantitative SWATH (Sequential Window Acquisition of All Theoretical fragment-ion spectra) based bottom-up proteomics. DEPs were identified by applying |Log_2_FC| ≥ 0.58, Q ≤ 0.05 cut-off filters and subsequently analyzed using Ingenuity Pathway Analysis (IPA) for pathway analysis. Volcano plots and curated pathway summaries (**Figures 3**-**5**) offered a comparative visualization of treatment responses. Functional annotation and molecular interpretation of the identified DEPs are consolidated in **Table 3**. The results revealed distinct yet interrelated mechanisms of action across the different treatment modalities that is discussed in the following subsections.

**Table 3:** The upregulation (red) and downregulation (blue) of the relevant differentially expressed proteins and their associated molecular pathways and mechanisms of action (Q ≤ 0.05) by N: UB at 3500 (3200:300 µM), Vinc at (0.94 µM), and their most potent combination N:UB: Vinc at 2450.94 (2240: 210: 0.94 µM) in the HKB-11 lymphoma cell line.

Treatment	Log_2_FC	*Gene ID*	Protein descriptions	Molecular pathway	Mechanism of action	Reference
N: UB (3500 µM)	6.5	*ITIH2*	Inter-alpha-trypsin inhibitor heavy chain H2	ECM organization, HA stabilization	Stabilizes the extracellular matrix (ECM) by binding to hyaluronic acid (HA), inhibiting tumor invasion and metastasis.	(53)
	4.6	*A2M*	Alpha-2-Macroglobulin	Protease inhibition, immune regulation	Inhibits proteases and modulates immune responses, suppressing tumor growth.	(54)
	6.0	*SERPINC1*	Serpin Family C Member 1	Ubiquitin-proteasome system, immune modulation	Induces apoptosis and inhibits M2 macrophage polarization, enhancing anti-tumor.	(55)
	3.2	*LTF*	Lactotransferrin	AKT signaling pathway, immune regulation.	Suppresses tumor growth by inhibiting AKT signaling and modulating immune responses.	(56)
	3.5	*GSN*	Gelsolin	Actin cytoskeleton organization and apoptosis regulation.	Regulates actin filament dynamics, affecting cell motility and apoptosis; its repression is associated with tumor progression.	(57)
	5.2	*ITIH3*	Inter-Alpha-Trypsin Inhibitor Heavy Chain 3	ECM organization, hyaluronan stabilization.	Similar to ITIH2, it contributes to ECM stability and inhibits metastasis.	(58)
	4.9	*C4BPA*	Complement Component 4 Binding Protein Alpha	Complement activation, immune response modulation.	Regulates the complement system, facilitating anti-tumor immunity by enhancing CD8+ T cell responses.	(59)
	−3.5	*VPS28*	Protein FAM136A	Endosomal Sorting Complex Required for Transport (ESCRT) Pathway	VPS28 is a component of the ESCRT-I complex, which is involved in endosomal sorting and membrane trafficking. Knockdown of VPS28 has been shown to suppress breast cancer cell proliferation and enhance apoptosis, potentially through the modulation of the ESCRT pathway and related signaling mechanisms.	(60)
	−2.8	*TMSB10*	Thymosin beta-10	PI3K-Akt Signaling Pathway, MAPK Signaling Pathway, Regulation of Actin Cytoskeleton	TMSB10 is involved in actin cytoskeleton organization and is overexpressed in various cancers. Its knockdown impairs cancer cell proliferation, migration, and invasion, suggesting that TMSB10 promotes tumor progression through these pathways.	(61)
	−3.0	*UBL5*	Ubiquitin-like protein 5	Fanconi Anemia (FA) DNA Repair Pathway	UBL5 interacts with FANCI to maintain the functional integrity of the FA DNA repair pathway. Downregulation of UBL5 impairs DNA repair mechanisms, leading to increased sensitivity of cancer cells to DNA-damaging agents, thereby inhibiting cancer progression.	(62, 63)
	−2.1	*RPL7L1*	Ribosomal protein uL30-like	Ribosome Biogenesis Pathway	RPL7L1 is a component of the 60S ribosomal subunit and is involved in protein synthesis. Overexpression of RPL7L1 has been observed in certain cancers, and its knockdown inhibits cancer cell proliferation and colony formation, indicating its role in tumor growth through the ribosome biogenesis pathway.	(64)
	−2.4	*RPS29*	Small ribosomal subunit protein uS14	Ribosomal Stress–MDM2–p53 Pathway	RPS29 is a component of the 40S ribosomal subunit. Deficiency in RPS29 activates the p53 pathway, leading to cell cycle arrest and apoptosis. This activation of the tumor suppressor p53 pathway contributes to inhibiting cancer progression.	(65)
Vincristine (0.94 µM)	3.8	*FGB*	Fibrinogen beta chain	Coagulation Cascade/Extracellular Matrix (ECM) Remodeling	FGB is a fibrinogen component that plays a role in blood clotting and ECM formation. Overexpression of FGB has been associated with tumor progression and metastasis. In renal cell carcinoma, downregulation of FGB by SIRT1 leads to inhibition of tumorigenesis, suggesting that elevated FGB levels may promote cancer progression.	(66)
	2.9	*FN1*	Fibronectin	Wnt/β-Catenin Signaling, Integrin-Mediated Signaling, Hippo Pathway	FN1 is a major ECM glycoprotein involved in cell adhesion, migration, and tissue repair. Its overexpression has been linked to tumor progression, metastasis, and therapy resistance in various cancers. FN1 can activate the Wnt/β-catenin pathway via β1 integrin, contributing to cisplatin resistance. FN1, derived from tumor-associated fibroblasts and macrophages, promotes tumor metastasis through the FN1-JUN-Hippo signaling pathway axis.	(67)
	1.1	*MAGOH*	Protein mago nashi homolog	PI3K/AKT Signaling, RAF/MEK/ERK Signaling, STAT3 Signaling	Knockdown disrupts mRNA processing and oncogenic signaling, causing cell cycle arrest and reduced metastasis.	(68)
	1.7	*NDUFB8*	NADH dehydrogenase [ubiquinone] 1 beta subcomplex subunit 8, mitochondrial	Mitochondrial Oxidative Phosphorylation (OXPHOS)	Inhibition impairs mitochondrial ATP production, increases ROS, and triggers apoptosis.	(69)
	−1.7	*POLR2A*	DNA-directed RNA polymerase II subunit RPB1	Transcriptional Regulation via RNA Polymerase II	Downregulation or inhibition of POLR2A impairs mRNA synthesis, leading to reduced protein production essential for cancer cell survival. In cancers with hemizygous deletion of POLR2A (e.g., colorectal and triple-negative breast cancers), further suppression sensitizes cells to transcriptional inhibitors like α-amanitin, resulting in selective tumor cell death.	(70, 71)
	−1.3	*NCDN*	Neurochondrin	MAPK/ERK Signaling Pathway	NCDN is involved in neuronal differentiation and synaptic plasticity. While its direct role in cancer is less characterized, downregulation may disrupt MAPK/ERK signaling, leading to decreased proliferation and increased apoptosis in certain cancer cell types.	(72)
	−2.2	*C4A*	Complement C4-A	Complement Activation via Classical and Lectin Pathways	C4A is a key component of the complement system, which can promote inflammation and tumor progression. Downregulation of C4A may reduce complement-mediated inflammation and the immunosuppressive tumor microenvironment, thereby inhibiting cancer progression.	(73)
	−1.3	*NUCKS1*	Nuclear ubiquitous casein and cyclin-dependent kinase substrate 1	PI3K/AKT/mTOR and SKP2-p21/p27 Axis	NUCKS1 functions as a transcriptional regulator promoting cell proliferation. Its downregulation leads to decreased SKP2 expression, accumulating cyclin-dependent kinase inhibitors p21 and p27 and causing cell cycle arrest. Additionally, suppressing NUCKS1 impairs PI3K/AKT/mTOR signaling, reducing tumor growth and metastasis.	(74)
	−1.1	*RRBP1*	Ribosome-binding protein 1	Endoplasmic Reticulum (ER) Stress Response, Hippo/YAP Signaling Pathway	RRBP1 is involved in protein translocation into the ER and is associated with ER stress responses. Its downregulation induces ER stress and activates the unfolded protein response, leading to apoptosis in cancer cells. Additionally, RRBP1 suppression affects the Hippo/YAP pathway, reducing chemoresistance and tumor growth.	(75)
N: UB: Vinc (2450.94 µM)	1.5	*SLC38A2*	Sodium-coupled neutral amino acid symporter 2	Amino Acid Transport/mTORC1 Signaling	SLC38A2 is an amino acid transporter that facilitates the uptake of glutamine and other neutral amino acids. It contributes to activating the mTORC1 pathway, which promotes cell growth and proliferation. Inhibiting SLC38A2 can disrupt amino acid homeostasis, leading to reduced mTORC1 signaling and suppression of tumor growth.	(76)
	1.5	*PNP*	Purine nucleoside phosphorylase	Purine Salvage Pathway/TLR Activation	PNP is involved in purine metabolism. Inhibition of PNP leads to the accumulation of deoxyguanosine, which can activate toll-like receptors (TLRs), enhancing immune responses against tumor cells. This immunostimulatory effect can contribute to cancer inhibition.	(77)
	1.4	*MAP1LC3B2*	Microtubule-associated proteins 1A/1B light chain 3 beta 2; Microtubule-associated proteins 1A/1B light chain 3B	Autophagy/Mitophagy	Upregulation of MAP1LC3B2 enhances autophagic activity, which can lead to the degradation of damaged organelles and proteins. This can potentially suppress tumor progression by maintaining cellular homeostasis.	(78)
	1.3	*GMNN*	Geminin	DNA Replication Control/Cell Cycle Regulation	GMNN inhibits DNA replication by preventing the assembly of the pre-replication complex. Upregulation of GMNN ensures proper cell cycle progression and prevents DNA re-replication, which can lead to genomic instability. GMNN may act as a tumor suppressor by maintaining genomic integrity in specific contexts.	(79)
	−2.9	*NNMT*	Nicotinamide N-methyltransferase	Methylation Metabolism/PP2A Activation/MAPK-Akt Signaling	Downregulation of NNMT activates the tumor suppressor PP2A, inhibiting the oncogenic MAPK/Akt signaling pathways. This results in decreased cancer cell proliferation and increased apoptosis.	(80)
	−2.1	*FGG*	Fibrinogen gamma chain	IL-6/STAT3 Signaling/Epithelial-Mesenchymal Transition (EMT)	The IL-6/STAT3 pathway regulates FGG. Downregulation of FGG leads to decreased expression of EMT markers such as Slug and ZEB1, reducing cancer cell migration and invasion.	(81)
	−2.5	*PLTP*	Phospholipid transfer protein	p53-Mediated Ferroptosis/Lipid Metabolism	PLTP is a target gene of the tumor suppressor p53. Downregulation of PLTP enhances p53-mediated ferroptosis, an iron-dependent form of cell death, thereby suppressing tumor growth.	(82)
	−2.2	*TARBP1*	Probable methyltransferase TARBP1	RNA Methylation/Gene Expression Regulation	TARBP1 is involved in RNA methylation processes. Downregulation of TARBP1 may disrupt aberrant RNA modifications, leading to decreased tumor cell proliferation and potential cancer inhibition.	(83)
	−2.0	*CYP4X1*	Cytochrome P450 4X1	CYP4X1 is involved in the metabolism of fatty acids and eicosanoids.	Downregulation of CYP4X1 may disrupt metabolic pathways that support tumor growth and metastasis, thereby inhibiting cancer progression.	(84)

#### 3.4.1 Dual therapy with N and UB 4:6 (3200: 300) induced structural reinforcement and immune reprogramming in HKB-11 lymphoma cells

The resulting network analysis, depicted in **Figure 3A**, highlighted several interconnected biological themes that suggested a coordinated cellular response to the treatment. A central and highly significant theme emerged around the modulation of cellular energy and nucleotide metabolism. Key proteins, including HSPD1, OGA, and DDX5, were implicated in the regulation of mitochondrial and tumor cell respiration. In contrast, others, such as CAB39L and FLCN, were linked to the biosynthesis of purine ribonucleotides and ATP.

Our proteomic analysis revealed a significant and coordinated downregulation of multiple DEAD-box (DDX) RNA helicases, suggesting that N: UB treatment may initiate a multi-pronged assault on RNA metabolism, which is essential for HKB-11 cell viability. Specifically, we observed a marked reduction in the nucleolar proteins DDX21 and DDX27, both of which are critical for rRNA processing and ribosome biogenesis (85). As cancer cells are highly dependent on elevated ribosome production to sustain their high proliferation rate, the simultaneous suppression of these helicases could severely impair the cell’s protein synthesis capacity, likely inducing ribosomal stress and subsequent cell cycle arrest. This assault on protein production is further compounded by the downregulation of DDX3X, a key helicase involved in translation initiation that is frequently overexpressed in cancers to drive the synthesis of oncogenic proteins (86). Furthermore, the reduction of DDX6, a central component of P-bodies involved in translational repression and mRNA decay, indicates a disruption of post-transcriptional gene regulation and cellular stress responses (87). Taken together, the concurrent downregulation of helicases governing ribosome assembly (DDX21, DDX27), translation initiation (DDX3X), and mRNA fate (DDX6) pointed to a catastrophic failure of the protein synthesis machinery, providing a potent and comprehensive mechanism for the anti-proliferative effects of N: UB on HKB-11 cells.

The analysis identified the down-regulated cyclin-dependent kinase 4 encoded by CDK4 (Log_2_FC= -0.698) as a key nodal protein regulating cell death, a process functionally tied to the observed alterations in ATP concentration and mitochondrial function. In addition, other cyclin-dependent kinases, including CDK6 and CDK1, were also significantly downregulated in HBK-11 cells upon N: UB treatment (Log_2_FC -1.07 and -1.06, respectively). A key finding from our proteomic analysis was the significant downregulation of Cyclin-Dependent Kinase 4 (CDK4) in HKB-11 cells following N: UB treatment. As a critical regulator of the G1-to-S phase transition, CDK4’s primary role is to phosphorylate and inactivate the retinoblastoma tumor-suppressor protein (Rb), thereby licensing cells to enter the cell cycle. The observed reduction in CDK4 protein levels strongly suggested that N: UB exerts its anti-proliferative effects by disrupting this canonical pathway, leading to a potential G1-phase cell cycle arrest. This mechanism is a cornerstone of modern anticancer therapies, where inhibiting the CDK4/6-Rb axis is a validated strategy for inducing a cytostatic response (88). This molecular insight directly supported our IPA network analysis, which highlighted the regulation of cell survival and death as a major biological theme influenced by CDK4 modulation, as shown in **Figure 3A**. It suggested the potential of the N: UB combination as a CDK1,4,6 inhibitor. Moreover, 313 proteins were significantly regulated in the HBK-11 cell lysate upon treatment with the N: UB combination compared to untreated cells (Q ≤ 0.05 and |Log_2_FC| ≥ 1), as listed in **Supplementary Table S1**.

Supporting these core metabolic and survival shifts, the proteomic data also pointed to significant regulation of fundamental cellular machinery. The network highlighted a theme of protein expression and transport, with entities like FMR1, H2AX, and IGF1R modulating protein synthesis and intracellular trafficking. These processes are likely orchestrated by upstream regulatory changes, as indicated by a fifth theme centered on cellular responses to gene expression. Here, regulatory proteins such as SMAD4, SMYD1, and SPEN were identified as influencing transcriptional activity. Collectively, these findings suggested that N: UB treatment induced a significant and integrated response in HBK11 cells, characterized by a shift in mitochondrial bioenergetics that consequently impacts cell survival pathways, all of which is underpinned by a reconfigured network of gene and protein expression.

The N: UB combination also modulated proteins associated with extracellular matrix (ECM) stability, immune modulation, and mitotic inhibition. The most significantly upregulated proteins included inter-alpha-trypsin inhibitor heavy chains ITIH2 and ITIH3 (Log_2_FC = 6.5 and 5.2, respectively), which stabilize hyaluronic acid and restrict ECM degradation, thereby suppressing tumor invasion and metastatic spread (53, 58). Additional upregulated regulators of the tumor microenvironment included alpha-2-macroglobulin (A2M; Log_2_FC = 4.6), which neutralizes proteases and inflammatory mediators, and C4BPA (Log_2_FC = 4.9) enhancing CD8+ T-cell responses and reducing complement-mediated tumor evasion (54, 59).

Further immune-relevant proteins such as SERPINC1 (Log_2_FC = 6.0) and lactotransferrin (LTF; Log_2_FC = 3.2) were upregulated, suggesting potential inhibition of M2 macrophage polarization and AKT pathway suppression, respectively (55, 56). Gelsolin (GSN; Log_2_FC = 3.5), known for its dual role in regulating actin dynamics and apoptosis, was also elevated, likely contributing to reduced motility and enhanced programmed cell death (57).

Simultaneously, tumor survival pathways were significantly suppressed. VPS28 (Log_2_FC = -3.5), part of the ESCRT-I complex involved in endosomal trafficking and proliferation, was strongly downregulated (60). UBL5 (Log_2_FC = -3.0), a key player in Fanconi anemia-mediated DNA repair, was diminished, impairing genomic maintenance and increasing sensitivity to DNA-damaging agents (62). Downregulation of TMSB10, RPL7L1, and RPS29 disrupts cytoskeletal integrity and ribosomal biogenesis, leading to ribosomal stress and activation of p53-mediated apoptosis (61, 65).

**Figure 3A** highlighted enriched ECM stabilization, immune modulation, and DNA repair inhibition pathways. The volcano plot **Figure 3B** demonstrated a balanced distribution of upregulated structural and immunomodulatory proteins and downregulated mitotic regulators, reflecting a dual mechanism of tumor microenvironment reinforcement and intrinsic proliferative arrest. The IPA-enriched canonical pathways, as shown in **Figure 3C** and reported in **Supplementary Table S2**, indicate that N: UB treatment strongly suppresses global translation, ribosome biogenesis, and protein targeting, while activating stress response pathways, including EIF2 signaling and mitochondrial functions. Moreover, this suggested a cellular state of proteotoxic and oxidative stress, which may lead to apoptosis or senescence.

The proteomic profiles of the N: UB treatment at 3500 µM (3200:300 µM) in this study was broadly consistent with our previous study (14), confirming the coordinated suppression of cell cycle regulators (CDK1, CDK4, CCNB1, CDCA8) and upregulation of ECM-stabilizing and immune-modulatory proteins (A2M, SERPINF1, ITIH2) that collectively impaired proliferation and invasiveness of HKB-11 lymphoma cells (15). Datasets in both studies highlighted enhanced mitochondrial activity and inflammatory signaling, indicating metabolic and immune reprogramming as central mechanisms. However, the current analysis extended these observations by uncovering a pronounced downregulation of DEAD-box RNA helicases (DDX21, DDX27, DDX3X, and DDX6), suggesting that ribosomal stress and translational arrest were additional anti-proliferative mechanisms not described in our previous study. Furthermore, the present data revealed stronger upregulation of immune effectors (SERPINC1, LTF, GSN) and broader inhibition of translation and ribosome biogenesis pathways, supporting a more comprehensive disruption of the tumor-supportive proteome. These differences may reflect the enhanced sensitivity of the current proteomic analysis or biological variability, but collectively reinforce the therapeutic potential of N: UB by targeting the cell cycle, metabolism, immune surveillance, and protein synthesis.

#### 3.4.2 Vinc monotherapy (0.94 µM) led to selective inhibition of transcription and mitochondrial modulation in HKB-11 lymphoma cells

Vinc induced a focused yet effective perturbation of transcriptional and mitochondrial homeostasis in HKB-11 lymphoma cells. Notably, POLR2A (Log_2_FC = -1.7), the catalytic core of RNA polymerase II, was significantly downregulated, suggesting impaired transcriptional activity and increased susceptibility to transcription inhibitors, particularly in cancers with partial POLR2A deletions (70). Suppression of NUCKS1 (Log_2_FC = -1.3) suggested disrupted SKP2–p21/p27 signaling, leading to G1 cell cycle arrest (74), while RRBP1 (Log_2_FC = -1.1) downregulation implicated ER stress and unfolded protein response activation, both linked to apoptosis induction (75).

Interestingly, the mitochondrial protein NDUFB8 (Log_2_FC = 1.7) was upregulated in the HKB-11 cells, reflecting a compensatory enhancement of oxidative phosphorylation under cytotoxic stress (69). Elevated expression of FGB and FN1, coagulation and ECM remodeling pathways components, may represent a stress-adaptive stromal response (66, 67).

**Figure 4A** underscored disrupted transcriptional regulation and mitochondrial stress signaling as dominant themes. The volcano plot, **Figure 4B**, exhibited a moderate yet distinct spectrum of DEPs, consistent with Vinc’s known mechanism of inducing mitotic catastrophe through spindle apparatus interference. The IPA-enriched canonical pathways, as shown in **Figure 4C** and reported in **Supplementary Table S2**, indicate that inc monotherapy may induced a complex cytotoxic phenotype involving elevated mitochondrial respiration, impaired mitochondrial quality control, suppressed immune cell cytotoxic programs, and accumulation of oxidative stress. These effects, acting together, promoted apoptosis and may partially explain the drug’s efficacy against HKB-11 lymphoma cells.

Canonical pathway enrichment, upstream regulator prediction, and disease-function annotations revealed consistent mitochondrial and proteostasis-related alterations across all conditions after treatment with Vinc (**Figure 5**).

Oxidative phosphorylation and respiratory electron transport were the most significantly upregulated pathways (Z-score > 2), indicating enhanced mitochondrial activity across all Vinc-containing treatments. The key proteins contributing to this enrichment included components of the electron transport chain (ETC), such as NDUFB3, COX5B, MT-ND3, ATP5MK, and VDAC1, 2, and 3. These proteins span complexes I, IV, and V of the ETC, suggesting a global upregulation of mitochondrial respiration. Enhanced oxidative phosphorylation has previously been associated with the accumulation of pro-apoptotic reactive oxygen species (ROS) in cancer cells undergoing therapeutic stress (89–91).

Conversely, the sirtuin signaling pathway was consistently inhibited across all Vinc monotreatments (Z-score < –2). Moreover, this was accompanied by the predicted downregulation of SIRT3, a mitochondrial deacetylase responsible for controlling oxidative stress and maintaining mitochondrial integrity (92). The inhibition of SIRT3 may sensitize lymphoma cells to mitochondrial dysfunction by impairing ROS detoxification and promoting pro-apoptotic signaling. Inhibition of sirtuin pathways, particularly SIRT3, has been shown to exacerbate mitochondrial stress and sensitize tumor cells to chemotherapeutic agents (93).

The selective autophagic clearance of aggregated proteins was also suppressed, suggesting impaired proteostasis and accumulation of damaged or misfolded proteins. The inhibition of this process is consistent with the observed suppression of the HSP90 chaperone signaling pathway, which plays a central role in folding and stabilizing oncogenic client proteins (94). Proteins such as HSP90AA1 and HSPB1 were downregulated, indicating collapse of the chaperone-mediated stress response. This disruption of proteostasis may potentiate apoptosis by overloading cellular quality control systems.

Several upstream regulators were identified as significantly activated across all three Vinc monotherapy conditions. CD38, a NADase that consumes intracellular NAD+, was among the top-ranked activators of the process. By reducing NAD+ availability, CD38 can suppress sirtuin activity, which aligns with the observed inhibition of sirtuin signaling (95). HGF (Hepatocyte Growth Factor) and VDR (Vitamin D Receptor) were also activated in the HKB-11 cells upon Vinc treatment. HGF is known to promote cell survival and proliferation, potentially representing a compensatory response to therapy-induced stress (96). Additionally, VDR activation has been linked to the modulation of oxidative stress and immune regulation, possibly reflecting a secondary stress-adaptive mechanism (97).

Common DEPs shared across multiple enriched pathways and upstream networks included MT-ND3, NDUFB3, COX5B, VDAC1/2/3, ATP5MK, HSP90AA1, and SIRT3. These proteins collectively contribute to mitochondrial function, redox regulation, and protein folding, reinforcing the central role of mitochondrial stress and proteotoxicity in the cytotoxic mechanism. Upregulation of oxidative phosphorylation, combined with the inhibition of sirtuin signaling, aggrephagy, and HSP90-mediated protein folding, promotes ROS accumulation, protein aggregation, and apoptosis. Taken together, these findings supported a coordinated model in which Vinc treatments disrupt mitochondrial and proteostasis homeostasis in lymphoma cells. These effects were further reinforced by upstream regulators that modulate mitochondrial metabolism, NAD+ availability, and responses to oxidative stress.

#### 3.4.3 Triple combination N: UB: Vinc at (2450.94 µM) induced synergistic disruption of metabolism, epigenetics, and cell cycle control in HKB-11 cells

The triple combination therapy of N: UB: Vinc at (2450.94 µM) elicited the most extensive proteomic reprogramming across biological pathways in HKB-11 cells. Upregulation of MAP1LC3B2 (Log_2_FC = 1.4) signaled enhanced autophagic flux and mitophagy, potentially contributing to cell death by eliminating dysfunctional organelles (78). GMNN (Log_2_FC = 1.3), an inhibitor of DNA re-replication, was elevated, supporting chromosomal stability during cytotoxic stress (79). SLC38A2 (Log_2_FC = 1.5) facilitated glutamine transport and maintained mTORC1 signaling under stress conditions (76), while PNP (Log_2_FC = 1.5) promoted purine salvage and TLR-mediated immune activation (77).

**Figure 6:**
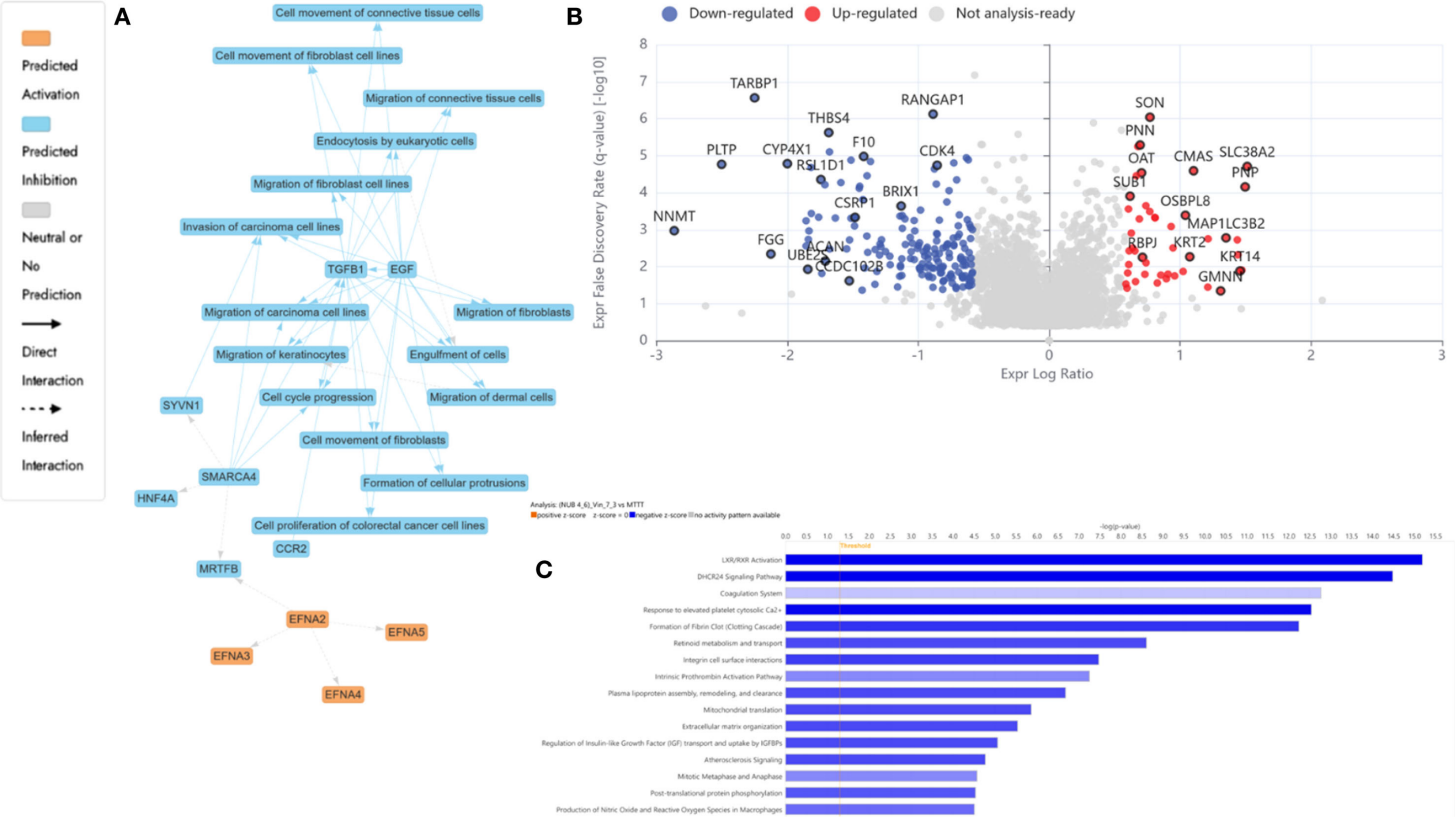
Proteomics analysis of N: UB: Vinc combination at 2450.94 µM (2240: 210: 0.94 µM) vs mono treatment in HKB-11 lymphoma cells, showing **(A)** IPA graphical summary showing two biological enriched themes. The significantly regulated proteins were identified (Q ≤ 0.05, Log_2_ fold change ≥ 0.58). **(B)** Volcano plot of significantly regulated proteins (absolute Log_2_ fold change ≥0.58 and Q ≤ 0.05). **(C)** Top IPA enriched canonical pathways (|Z| >2 and Q <0.05).

Among the most strongly downregulated proteins was NNMT (Log_2_FC = -2.9), which modulates NAD+ metabolism and supports the MAPK/Akt pathway. Its suppression reactivates PP2A and induces apoptosis (80). PLTP (Log_2_FC = -2.5) downregulation can promote p53-mediated ferroptosis, an emerging form of programmed cell death dependent on iron metabolism (82). Additional suppression of TARBP1 (Log_2_FC = -2.2) and CYP4X1 (Log_2_FC = -2.0) reflected potential epigenetic destabilization and disruption of tumor-supportive lipid metabolism (83, 84). Beyond its pro-apoptotic effects, our findings suggested that ferroptosis may play a complementary role in HKB-11 cell death by the N: UB: Vinc combination. Ferroptosis is an iron-dependent, non-apoptotic pathway characterized by lipid peroxidation and oxidative membrane damage (98). This pathway has garnered attention as a therapeutic target because many cancers, particularly those resistant to apoptosis, remain susceptible to ferroptosis (99). The observed increase in ROS and potential disruption of redox homeostasis in our study could reflect ferroptotic activity. Inducing ferroptosis may enhance treatment efficacy by overcoming resistance to classical chemotherapeutics. Recent evidence suggested that microtubule-targeting agents, including vinca alkaloids, can indirectly promote ferroptosis through mitotic stress and metabolic perturbations (100). Therefore, the enhanced cell death observed at intermediate Vinc concentrations might involve a synergy between apoptosis and ferroptosis pathways. Targeting both apoptosis and ferroptosis could represent a promising strategy for enhancing lymphoma therapy. Assessing ferroptosis-specific markers, such as lipid ROS accumulation, iron overload, GPX4 downregulation, and ACSL4 upregulation, in future studies will provide insights into the molecular underpinnings of the combination. **Figure 6A** highlights tightly interconnected modules encompassing ferroptosis, autophagy, metabolic stress, and transcriptional shutdown. The corresponding volcano plot **Figure 6B** confirmed the most comprehensive DEP distribution observed across all treatment conditions, validating the triple combination’s synergy and multidimensional cytotoxicity. The IPA-enriched canonical pathways, as shown in **Figure 6C** and reported in **Supplementary Table S2**, indicate that the synergistic combination at a dosage of 2450.94 µM imposes a multifaceted inhibitory profile on pro-tumor metabolic, vascular, and proliferative programs, contrasting with the stress-induced mitochondrial activation seen in monotherapy. Specifically, the suppression of lipid metabolism (LXR/RXR), coagulation, ECM remodeling, and mitotic checkpoints revealed potential tumor-suppressive reprogramming unique to the combination.

**Figure 7:**
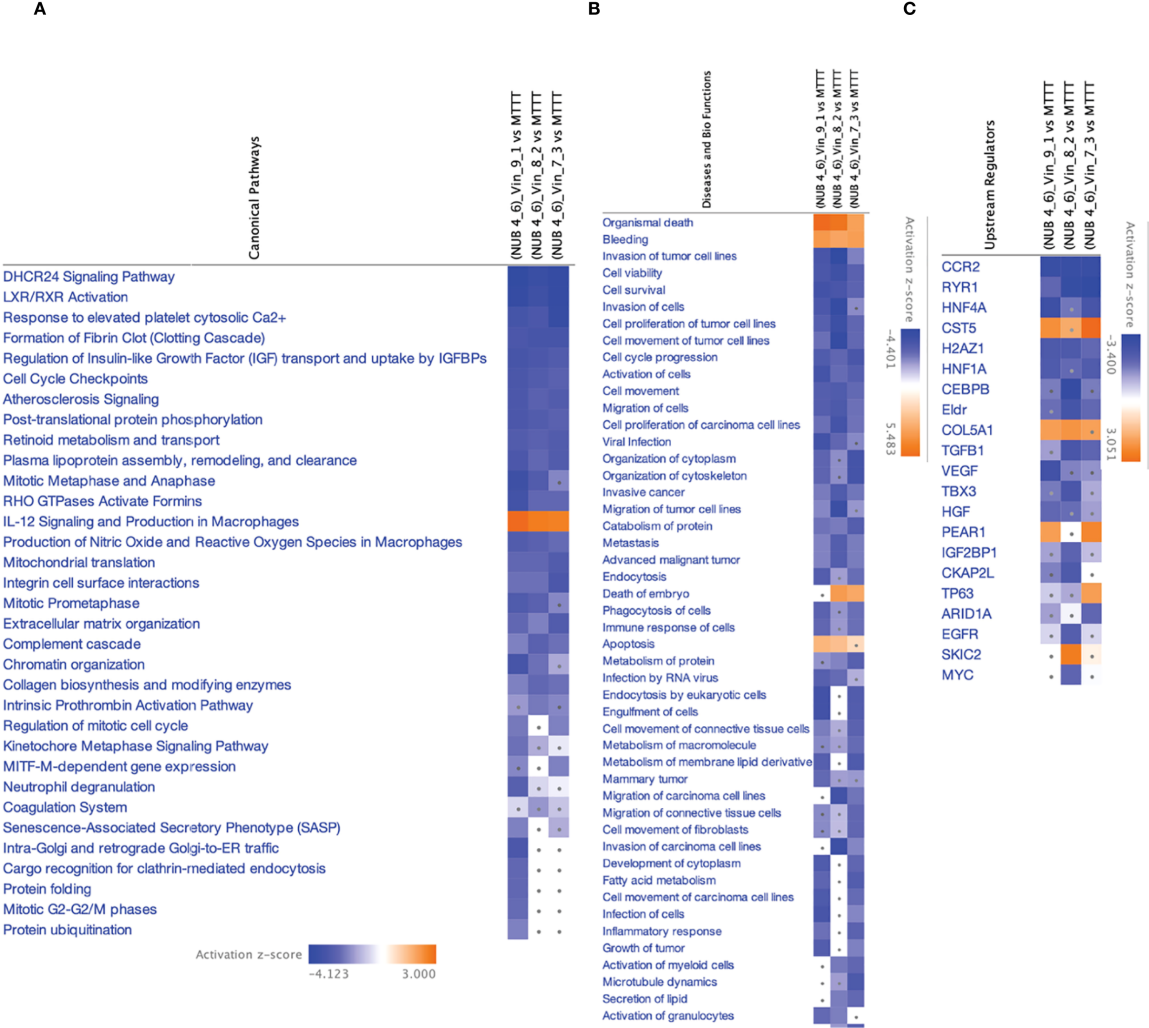
IPA analyses summary of the NUB (4:6) combinations with Vinc at (2450.94, 2800.63 and 3150.31 μM) against HKB-11 lymphoma cells using Differentially expressed proteins (DEPs) (|Z-score|>1 and Q <0.01), showing **(A)** Significant canonical pathways (|Z-score|>1 and Q <0.01), **(B)** Enriched diseases and biofunctions (|Z-score|>2.5 and Q <0.01) and **(C)** Significant upstream regulators (|Z-score|>2 and Q <0.01). The dots indicate absolute z-scores of less than 2.

IPA analysis of the proteomic profiles comparing triple treatment combinations of N, UB, and Vinc (2450.94, 2800.63 and 3150.31 μM) against monotherapies revealed widespread suppression of cancer-associated pathways in HKB-11 lymphoma cells (**Figures 7A–C**). The top five canonical pathways and upstream regulators significantly modulated by the combinations were primarily involved in cell cycle control, extracellular matrix remodeling, lipid metabolism, and pro-survival signaling.

##### 3.4.3.1 Cell cycle arrest and mitotic suppression

Multiple mitosis-related pathways, including cell cycle checkpoints, mitotic metaphase and anaphase, mitotic prometaphase, and regulation of mitotic cell cycle, were consistently downregulated (Z-score < –2). These pathways regulate the G_2_/M transition, chromatid alignment, and mitotic spindle function, all of which are critical for proliferation in rapidly dividing lymphoma cells. The suppression of these checkpoints likely reflects Vinc’s microtubule-targeting effects and the enhancement of mitotic arrest by the combination therapy. Similar mitotic dysregulation has been reported as a primary mechanism of Vinc-induced apoptosis in hematologic malignancies (101).

##### 3.4.3.2 Inhibition of ECM and invasion pathways

Pathways governing cell adhesion and matrix interaction, including integrin cell surface interactions, ECM organization, and collagen biosynthesis, were strongly suppressed. These ECM components are essential for lymphoma dissemination and interaction with the tumor microenvironment. Disruption of integrin-mediated signaling reduces lymphoma invasiveness and impairs matrix remodeling (102). Correspondingly, IPA disease-function enrichment revealed significant inhibition of cell invasion, migration, and tumor progression signatures.

##### 3.4.3.3 Downregulation of lipid metabolism and LXR/RXR signaling

Both LXR/RXR activation and the DHCR24 signaling pathway were among the most suppressed canonical pathways. LXR/RXR receptors are master regulators of cholesterol metabolism, inflammation, and immune suppression in cancer (103). DHCR24, an enzyme involved in cholesterol synthesis, has also been linked to therapy resistance and anti-apoptotic activity in leukemia (104). Their downregulation may contribute to impaired metabolic flexibility and enhanced susceptibility to treatment-induced stress in lymphoma cells.

##### 3.4.3.4 Suppression of IGF signaling axis

The pathway governing the regulation of Insulin-like Growth Factor (IGF) transport and uptake by IGFBPs was consistently inhibited across all combinations (2450.94, 2800.63 and 3150.31 μM). IGF signaling drives proliferation and resistance to apoptosis in B-cell and T-cell lymphomas via the PI3K/AKT and MAPK pathways (105). The observed downregulation of IGF2BP1, a key RNA-binding protein stabilizing IGF transcripts, supported the inhibition of this survival axis and potentiated the pro-apoptotic effects of the combination.

##### 3.4.3.5 Activation of residual pro-survival signaling: HGF and VEGF

Despite the broad suppression of oncogenic pathways, IPA upstream regulator analysis revealed activation of HGF (Hepatocyte Growth Factor) and VEGF (Vascular Endothelial Growth Factor) in the 2800.63 μM combination. These regulators promote angiogenesis, cell survival, and matrix remodeling hallmarks of lymphoma progression and relapse. Their enrichment may represent a compensatory response to the overwhelming cytotoxic stress induced by the triple combination (105). However, their activation did not appear sufficient to override the broader tumor-suppressive signaling landscape in the HKB-11 cells.

Collectively, these findings revealed that the N: UB: Vinc combination exerted its anti-lymphoma effects through a coordinated disruption of key oncogenic pathways, including cell cycle progression, matrix remodeling, metabolic homeostasis, and growth factor signaling in the HKB-11 cells. This broad-spectrum suppression contrasted with the more focused mitochondrial stress response observed in Vinc monotherapy, underscoring the mechanistic basis of the observed synergistic cytotoxicity.

#### 3.4.4 Functional synthesis of DEPs across treatments in the HKB-11 lymphoma cells

Proteomic and IPA analyses revealed that Vinc monotherapy and the N: UB: Vinc combination at 2450.94 μM elicited mechanistically distinct anti-lymphoma responses in the HKB-11 cells. Vinc alone induced a focused cytotoxic profile characterized by mitochondrial oxidative phosphorylation activation, transcriptional suppression, and modest ECM remodeling. Notably, the upregulation of NDUFB8 suggested enhanced mitochondrial respiration and ROS production, a known pro-apoptotic mechanism under chemotherapeutic stress. Downregulation of POLR2A, the core subunit of RNA polymerase II, implied impaired transcriptional activity and heightened sensitivity to transcriptional inhibitors. The suppression of NUCKS1, a transcriptional activator of SKP2, suggested inhibition of the SKP2–p21/p27 axis and cell cycle arrest at G_1_. Moreover, the reduced expression of RRBP1, a key regulator of ER homeostasis, implicated the activation of the unfolded protein response and apoptotic signaling. The elevated levels of FGB and FN1, proteins involved in coagulation and ECM remodeling, may represent a reactive stromal remodeling response, potentially facilitating tumor survival under stress.

In contrast, the N: UB: Vinc combination induced a more multifaceted anti-cancer program that can potentially suppress tumor-promoting processes across metabolic, epigenetic, and immunological axes. Upregulation of MAP1LC3B2 and GMNN indicated induction of mitophagy and replication checkpoint control, contributing to cellular quality control and genomic integrity under cytotoxic stress. The combination also significantly downregulated NNMT, PLTP, and CYP4X1, thereby disrupting NAD^+^ metabolism, lipid transport, and cytochrome P450 activity, and sensitizing cells to p53-mediated ferroptosis, while impairing tumor-supportive metabolic flexibility. The reduced expression of TARBP1 suggested interference with aberrant RNA methylation and transcriptional regulation, thereby further limiting the expression of oncogenic genes. Additionally, upregulation of PNP and SLC38A2 suggested potential enhancement of purine salvage and glutamine transport, respectively, which may contribute to metabolic exhaustion and TLR-mediated immune activation. Notably, the downregulation of FGG, a STAT3-regulated coagulation factor, implied suppression of epithelial-mesenchymal transition (EMT) and reduced invasiveness.

Overall, the combination treatment outperformed Vinc monotherapy by coordinating the disruption of key oncogenic circuits. While Vinc targeted mitotic structures and induced mitochondrial stress, the addition of N and UB extended this effect to encompass the induction of ferroptosis, epigenetic destabilization, immune reactivation, and inhibition of matrix remodeling. These findings suggest that postbiotic co-therapy can convert Vinc’s focused cytotoxicity into a systems-level tumor-suppressive state, providing a mechanistic basis for the observed synergy and a rationale for advancing this combination in preclinical lymphoma models.

## 4 Conclusion, limitations, and future directions

This study systematically evaluated the antiproliferative and synergistic efficacy of the triple combination of N, UB, and Vinc against human lymphoma cells. Cell viability assays demonstrated strong synergistic activity at optimized concentrations (2450.94, 2800.63, and 3150.31 µM), achieving up to 99.9% inhibition of lymphoma cell growth (HKB-11 and Hs 313.T) with reduced toxicity toward normal stromal HS-5 cells. Reactive oxygen species (ROS) analysis revealed marked oxidative stress induction, particularly at the highest triple dose, suggesting a role for oxidative damage in the observed cytotoxicity. Flow cytometric apoptosis assays confirmed significant increases in both early and late apoptotic populations, with pronounced effects across all tested concentrations of Vinc. These findings suggest that the combination is effective in inducing apoptotic cell death through both oxidative and non-oxidative mechanisms.

Proteomic profiling by LC-MS/MS provided molecular insights into the mechanisms underlying these effects, identifying several proteins that were significantly differentially expressed (|Log_2_FC| ≥ 0.58, Q ≤ 0.05). The combination upregulated proteins involved in autophagy (MAP1LC3B2), cell cycle regulation (GMNN), and immune modulation (PNP), while downregulating key oncogenic and metabolic regulators, including NNMT, PLTP, CYP4X1, and TARBP1. These data suggest that the combination modulates multiple pathways associated with tumor growth, metabolism, and immune responses.

The proteomic profiling presented here provides an exploratory map of candidate molecular regulators, including MAP1LC3B2, GMNN, and NNMT, which may underlie the observed synergistic effects. While the functional validation of these proteins was beyond the scope of the current study, future work can focus on targeted knockdown or inhibition studies, as well as validation in 3D organoids and *in vivo* lymphoma models, to establish direct causal links between changes in protein expression and therapeutic outcomes.

This study has limitations. All findings were derived from *in vitro* models, which do not fully replicate the tumor microenvironment, drug metabolism, or immune interactions present *in vivo*. While the low-to-mid concentrations tested demonstrated selectivity and are closer to achievable *in vivo* levels, the highest concentrations exceeded physiologically relevant thresholds. They should be regarded as proof of concept rather than a clinically translatable finding. Furthermore, the pharmacokinetics and bioavailability of N and UB remain poorly defined. N, as a peptide, exhibits low oral bioavailability and is prone to enzymatic degradation, although encapsulation and chemical modifications have been proposed to enhance its stability (106). UB, has been detected in human plasma at nanomolar to low micromolar levels following dietary intake (107). In contrast, Vinc is an approved chemotherapeutic with known pharmacokinetics, achieving plasma concentrations of 10–100 nM in patients (30, 38). Future research should address these limitations. *In vivo* studies using animal models and organoids are required to validate the efficacy, selectivity, and safety of the combination under physiologically relevant conditions. Furthermore, pharmacokinetic and pharmacodynamic analyses should be conducted to establish the feasibility of achieving therapeutic concentrations of N and UB, as well as to optimize dosing regimens. Advanced drug delivery systems, including nanoparticles and prodrug approaches, warrant exploration to improve the stability and bioavailability of N and UB. Further mechanistic investigations are needed to elucidate additional pathways involved in selectivity, including possible ferroptotic and immunomodulatory effects. Finally, identifying predictive biomarkers of response could inform patient stratification strategies in future clinical trials.

These findings provide a mechanistic rationale for combining gut microbial metabolites with conventional chemotherapy to enhance efficacy and selectivity against lymphoma. Robust preclinical and translational studies are crucial for advancing this approach toward clinical application.

## Data Availability

The mass spectrometry proteomics data have been deposited in the ProteomeXchange Consortium via the PRIDE (108) partner repository with the dataset identifier PXD065631.
